# Characterization and Structural Insights of the Reaction Products by Direct Leaching of the Noble Metals Au, Pd and Cu with *N*,*N*′-Dimethyl-piperazine-2,3-dithione/I_2_ Mixtures

**DOI:** 10.3390/molecules26164721

**Published:** 2021-08-04

**Authors:** Angela Serpe, Luca Pilia, Davide Balestri, Luciano Marchiò, Paola Deplano

**Affiliations:** 1 Department of Civil and Environmental Engineering and Architecture, INSTM Unit, University of Cagliari, Via Marengo 2, 09123 Cagliari, Italy; 2 Environmental Geology and Geoengineering Institute of the National Research Council (IGAG-CNR), Via Marengo 2, 09123 Cagliari, Italy; 3 Department of Mechanical, Chemical and Materials Engineering, University of Cagliari, Via Marengo 2, 09123 Cagliari, Italy; pilialuc@unica.it; 4 Department of Chemical, Life and Environmental Sustainability Sciences, University of Parma, 43124 Parma, Italy; davide.balestri@studenti.unipr.it; 5 Department of Chemical and Soil Sciences, University of Cagliari, Monserrato, 09042 Cagliari, Italy

**Keywords:** precious metals, gold, palladium, copper, recycling, dithiones, iodine, X-ray diffraction, DFT calculations

## Abstract

In the context of new efficient and safe leaching agents for noble metals, this paper describes the capability of the Me_2_pipdt/I_2_ mixture (where Me_2_pipdt = N,N′-dimethyl-piperazine-2,3-dithione) in organic solutions to quantitatively dissolve Au, Pd, and Cu metal powders in mild conditions (room temperature and pressure) and short times (within 1 h in the reported conditions). A focus on the structural insights of the obtained coordination compounds is shown, namely [AuI_2_(Me_2_pipdt)]I_3_ (**1**), [Pd(Me_2_pipdt)_2_]I_2_ (**2a**) and [Cu(Me_2_pipdt)_2_]I_3_ (**3**), where the metals are found, respectively, in 3+, 2+ and 1+ oxidation states, and of [Cu(Me_2_pipdt)_2_]BF_4_ (**4**) and [Cu(Me_2_dazdt)_2_]I_3_ (**5**) (Me_2_dazdt = *N*,*N*′-dimethyl-perhydrodizepine-2,3-dithione) compared with **3**. Au(III) and Pd(II) (*d^8^* configuration) form square–planar complexes, whereas Cu(I) (*d*^10^) forms tetrahedral complexes. Density functional theory calculations performed on the cationic species of **1**–**5** help to highlight the nature of the bonding in the different complexes. Finally, the valorization of the noble metals-rich leachates is assessed. Specifically, gold metal is quantitatively recovered from the solution besides the ligands, showing the potential of these systems to promote metal recycling processes.

## 1. Introduction

**Scheme 1:**
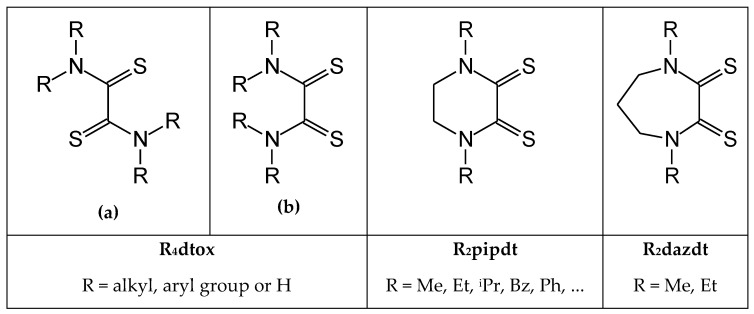
Acyclic and cyclic hexa- and hepta-atomic dithiooxamides.

Increasing attention is currently devoted towards effective and sustainable agents for noble metal (NM) recovery from secondary sources, due to the scarcity of accessible natural reserves of these metals which represent crucial elements for a great number of industrial applications (high-tech equipment, jewelry, catalysis, etc.) [1,2,3]. In this context, stimulated by world-wide regulations aiming for a more respectful approach towards the environment and natural resources, several classes of organic ligands have been investigated in the last three decades as potential alternatives to the harmful and polluting cyanides and aqua-regia lixiviants conventionally employed at industrial level for the hydrometallurgical reclamation of NMs [4,5,6,7,8,9]. Among them, thiourea and thiourea derivatives have long been recognized as versatile and effective complexing agents, finding application in a variety of fields. Due to their peculiar affinity towards *soft* metal ions (accordingly to the Hard–Soft Acid and Base Lewis theory), they have been demonstrated to be suitable candidates as NM leaching agents when in the presence of appropriate oxidizing agents. For example, thiourea acidic solutions in the presence of Fe^3+^ ions (as well as H_2_O_2_ and O_3_), has proved to be employable, including at the industrial level, for both chemical extraction of gold from ores and for chemical etching of gold and gold alloys [10,11,12]. More recently thiourea leaching has been employed in NM recovery from waste printed circuit boards [13,14]. Numerous advantages are related to the use of thiourea-based leaching systems, namely efficiency, low environmental impact, cheap reagents, mild experimental conditions, even if some criticisms are still presently preventing their wide-spread use in industrial applications: first of all, the high loss of non-recyclable reagents due to low selectivity and ligand degradation and oxidation. Beside thiourea, other thiourea derivatives namely dithioxamides (DTOs), demonstrated their powerful action as NM leaching agents when in the presence of oxidizing agents like *soft* halogen/interhalogens [15]. Despite the similarity with thiourea, they demonstrated stability to oxidation and reduction phenomena limiting degradation and increasing the recyclability of the ligand. Furthermore, DTO may give chelation through S-donor atoms and, when *d*^8^ *soft* metal ions are formed, provide their preferred square –planar geometry. Several examples of NM leaching processes based on the use of organic solutions of cyclic ad acyclic DTO combined with iodine (and IBr), have been reported in the literature [16,17,18,19,20,21,22,23,24] and patented for industrial application in the effective and sustainable recovery of NMs contained in WEEE (waste electrical and electronic equipment) [20] and in end-of-life three way catalysts [23]. These reagents, with combined complexing and oxidizing properties, demonstrated high efficiency and very low environmental impact, even if they are mostly not available on the market. A variety of NMs complexes were obtained and isolated from the leaching reaction depending on the metal and the DTO-ligand/halogen mixture employed [15]. Indeed, DTOs have demonstrated their versatility due to the possible configurations and electronic distributions they can assume when chelating the metal, giving the formed complexes peculiar properties. More specifically, as shown in Scheme 1, in acyclic DTO molecules with NR_2_ substituents in the α-dithione moiety, a twisted conformer (**a**) about the central C–C bond is favored, while a planar one (**b**) is hindered for steric reasons [25].

**Scheme 2:**
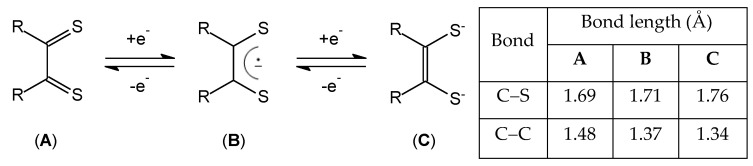
Reversible redox processes of α-dithiones and typical bond distances on the C_2_S_2_ moiety [26].

When these molecules work as S,S-chelating ligands to a metal, planarity inside the thioamide system is generally preserved, while planarity inside the M(S_2_C_2_)_2_ pentatomic ring is rarely observed. Reaching planarity will make DTO comparable with α-dithiones (see Scheme 2), the readily reducible ligand systems in the so-called class of dithiolene complexes able to undergo reversible redox processes which involve the filling of the four frontier orbitals of π-symmetry (the first two occupied in dithioketone, the third in ene-1,2-dithiolate) [26].

It has been established that electron-withdrawing substituents (CN) decrease the energy of these orbitals making difficult for the ene-1,2-dithiolates to undergo oxidation to reach the dithione form (**A**). On the contrary, electron-donating NR_2_ substituents increase the energy of these frontier orbitals making the reduction of DTO difficult, hence limiting the access to the reduced forms **B** and **C**. In summary, steric and electronic factors hinder the ability of DTO ligands in various complexes to behave as dithiolenes.

Here we will describe the capability of the Me_2_pipdt/I_2_ system in organic solutions, where Me_2_pipdt is the hexatomic cyclic DTO *N*,*N*′-dimethyl-piperazine-2,3-dithione (Scheme 1), to work as a powerful leaching agent towards Au, Pd, and Cu powders in mild conditions and short times. [Au^III^I_2_Me_2_pipdt]I_3_ (**1**), [Pd^II^(Me_2_pipdt)_2_]I_6_ (**2**) and [Cu^I^(Me_2_pipdt)_2_]I_3_ (**3**) coordination compounds were isolated in satisfactory yields as the main leaching products. The coordination behavior of the ligand is invariably S,S bidentate, but **1** and **2** comprise square–planar *d*^8^ metal Au(III) and Pd(II) complexes, while **3** involves a tetrahedrally coordinated *d*^10^-Cu(I) [27,28]. Additional results have shown that the 1+ oxidation state and the tetrahedral geometry are the preferred ones for copper complexes with these ligands. Indeed [Cu(Me_2_pipdt)_2_]BF_4_ (**4**), bearing the same cation of **3**, is obtained also when Me_2_pipdt was allowed to react with a copper(II) salt in the presence of NH_4_BF_4_. [Cu(Me_2_dazdt)_2_]I_3_ (**5**), the corresponding salt of **3** where the ligand consists in a hepta-atomic cyclic DTO, is similarly obtained by using as leaching mixture *N*,*N*′-dimethyl-perhydrodiazepine-2,3-dithione (Me_2_dazdt, Scheme 1)/I_2_. Detailed inspection of structural features of **1**–**5**, including the packing mode at the solid state, will be presented here with the view to provide information on bonding inside the cations and anions and interactions at supramolecular level, which may affect the formation of the different complexes and the effectiveness of noble metal dissolution [29]. Density functional theory (DFT) calculations, combined with experimental results, will help in elucidating the structure/bonding relationship. Lastly, a short overview on perspectives for using the obtained leaching products is discussed.

## 2. Results and Discussion

### 2.1. Leaching Behavior and Characterization of the Leaching Products

**Scheme 3:**
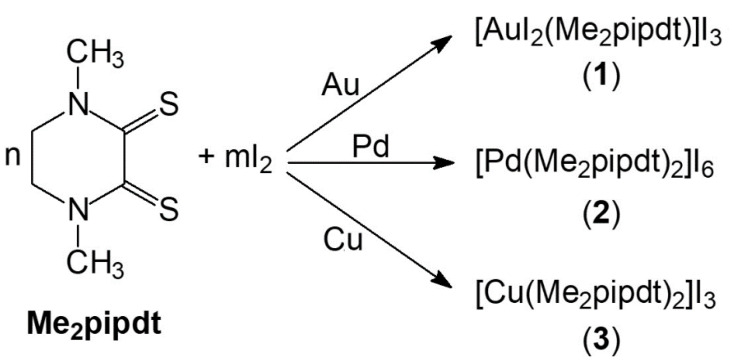
Reaction scheme for gold, palladium and copper powder dissolution by Me_2_pipdt/I_2_ mixtures. Reaction conditions: THF or CH_3_CN as solvent; room temperature; stirring. Complete leaching time: 1 h; Stoichiometry of product crystallization: (**1**) n = 1, m = 2.5; (**2**) n = 2, m = 3; (**3**) n = 2, m = 1.5.

As shown in Scheme 3, by reacting Me_2_pipdt/I_2_ mixtures with Au, Pd and Cu as metal powders in organic solvents (such as THF and CH_3_CN) under stirring at room temperature, almost clear solutions were obtained with a quantitative leaching of the metals in short times (within 1 h in the described conditions). Leaching experiments were performed at least three times for each metal on different amounts of metal powder and the yields of dissolution were found invariably quantitative in the reported times. [AuI_2_(Me_2_pipdt)]I_3_ (**1**), [Pd(Me_2_pipdt)_2_]I_6_ (**2**) and [Cu(Me_2_pipdt)_2_]I_3_ (**3**) were isolated as crystals in satisfactory yields (from 50% to almost quantitative) and fully characterized as detailed in Section 3.1.

When [Me_2_pipdtH]I_3_ salt [15] was used as the leaching agent, the same products were obtained. However, the use of Me_2_pipdt/I_2_ mixtures in the appropriate ratios improves time- and cost-effectiveness, since intermediate steps can be skipped out. X-ray crystal structure were solved for **1** and **3**. Well-formed crystals of **2** suitable for X-ray diffractometric studies, were obtained after several recrystallization experiments in form of **2a** compound, a salt of the same cationic complex where the counter-ions are iodides.

To better highlight the factors affecting the formation of the copper(I) complex in **3**, an attempt to prepare the corresponding Cu(II) complex [Cu(Me_2_pipdt)_2_]^2+^ in the form of BF_4_^−^ salt, avoiding the presence of the reducing I^−^ ions, was made. Accordingly, copper(II) chloride was reacted with Me_2_pipdt in a 1:2 molar ratio (in THF, under reflux in Ar atmosphere) in the presence of NH_4_BF_4_. The solution turned from light- to dark-green to purple, and the reaction went off in around 1 h. Following slow evaporation of THF and EtOH addition, crystals characterized as [Cu(Me_2_pipdt)_2_]BF_4_ (**4**) were obtained. It can be assumed that a copper(II) cationic complex is formed in solution (the green intermediate observed in this reaction) and that it undergoes spontaneous reduction to the more stable copper(I) complex, as earlier observed for similar compounds [27,28]. Copper(I) complexes with thione donors are not uncommon and those coordinated to biologically relevant ligands such as *N*,*N*′-dimethylimidazole-thione or others, are of interest to elucidate how coordination to sulfur and selenium inhibits copper-mediated oxidative damage. It has been shown that, when bound to Cu^+^, thione (and selone) ligands protect Cu^+^ from oxidation [30,31].

For comparison purposes, the leaching reaction of copper metal was also performed in the same experimental conditions by employing the Me_2_dazdt·2I_2_ adduct, in the 2:1 molar ratio with the metal powder. Copper metal was quantitatively dissolved in a short time at room temperature and the final product [Cu(Me_2_dazdt)_2_]I_3_ (**5**) was crystallized in an almost quantitative yield and fully characterized.

### 2.2. Molecular Structures

**Figure 1:**
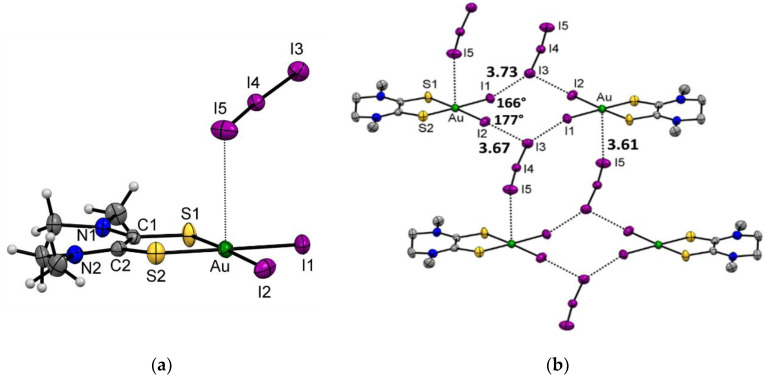
(**a**) Molecular structure of complex [Au(Me_2_pipdt)I_2_]I_3_ (**1**) with thermal ellipsoids plotted at the 50% probability level. (**b**) Portion of the crystal packing, showing intermolecular interactions as dashed bonds. Selected interaction distances (Å) and angles are indicated.

**Figure 2:**
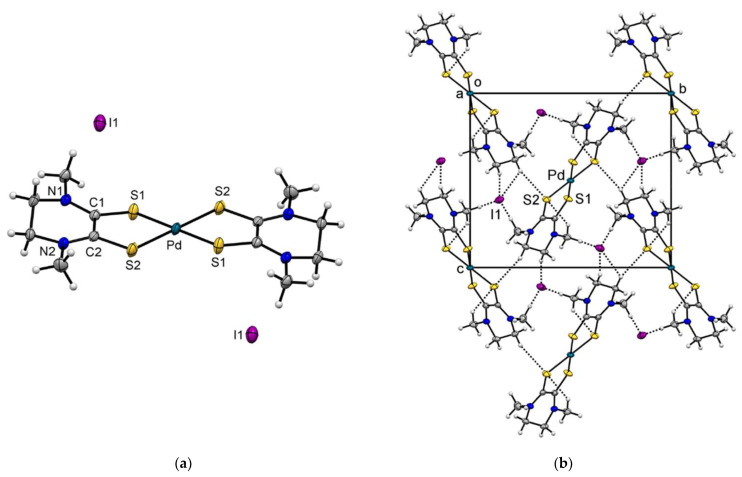
(**a**) Molecular structure of complex [Pd(Me_2_pipdt)_2_](I)_2_ (**2a**) with thermal ellipsoids plotted at the 50% probability level. (**b**) Portion of the crystal packing, showing intermolecular interactions as dashed bonds.

**Figure 3:**
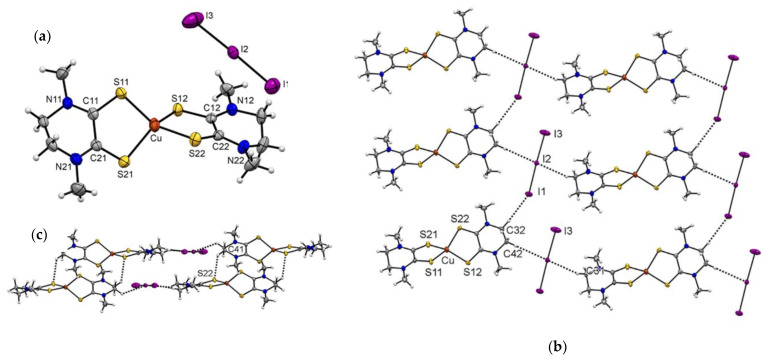
(**a**) Molecular structure of complex [Cu(Me_2_pipdt)_2_]I_3_ (**3**) with thermal ellipsoids plotted at the 50% probability level. (**b**,**c**) Portions of the crystal packing, showing intermolecular interactions as dashed bonds.

**Figure 4:**
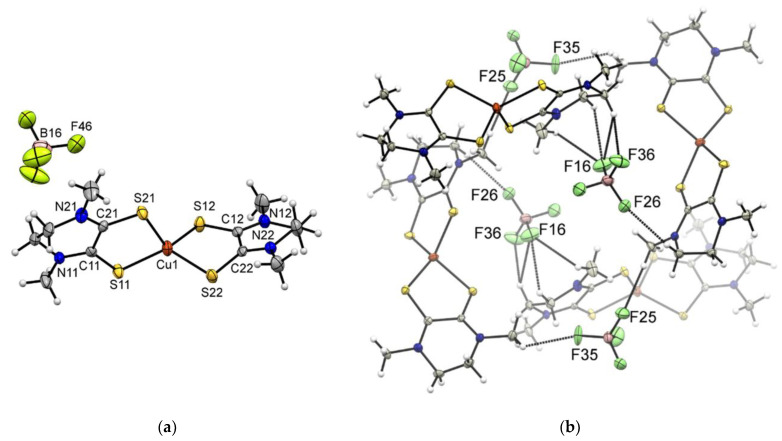
(**a**) Molecular structure of complex [Cu(Me_2_pipdt)_2_]BF_4_ (**4**) with thermal ellipsoids plotted at the 50% probability level. One molecular entity of the two comprising the asymmetric unit is reported for clarity. (**b**) Portion of the crystal packing, showing intermolecular interactions as dashed bonds.

**Figure 5:**
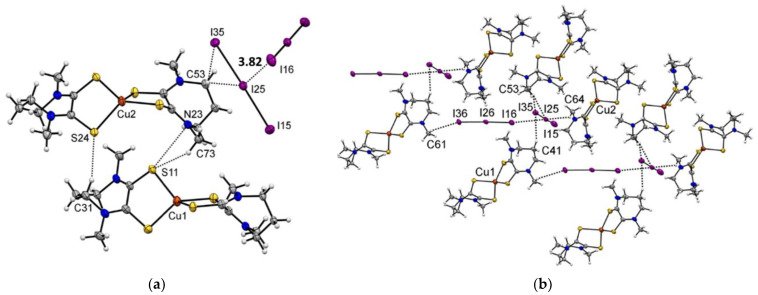
(**a**) Molecular structure of complex [Cu(Me_2_dazdt)_2_]I_3_ (**5**) with thermal ellipsoids plotted at the 50% probability level. (**b**) Portion of the crystal packing. Intermolecular interactions are depicted as dashed bonds. Halogen bond interaction is reported in Å.

Figure 1, Figure 2, Figure 3, Figure 4 and Figure 5 report the molecular structures of the obtained complexes (see Table S1 for the crystallographic data). The structures can be divided into two groups, one comprising *d*^8^ metal ions such as Au(III) and Pd(II), and the second group with a *d*^10^ cation, Cu(I). Indeed, despite the coordination behavior of the ligand is invariably S,S bidentate, with the former group square–planar complexes are formed, whereas in the latter group the complex geometry is tetrahedral.

In the gold complex [Au(Me_2_pipdt)I_2_]I_3_ (**1**), the Au-I bond distances are 0.3 Å longer than the Au-S ones, as expected for the larger dimension of the iodine anion with respect to the sulfur atom (Figure 1, Table 1).

In **1**, the crystal packing shows interesting features related to the presence of iodine atoms and the non-homogeneous distribution of the electron density on the I_3_^−^ molecular surface. Indeed, the iodine atom is prone to give rise to halogen bonds according to the high polarizability of its electron density. Specifically, when the iodine atom is bound to electron-withdrawing moieties, a portion of positive charge forms on the opposite side of the σ-bond (known as σ-hole), together with a portion of negative charge in a region perpendicular to the σ-bond (negative corona) [32,33]. Interestingly, this non-homogeneous distribution of the electron density is also present in the I_3_^−^ anion, with the σ-hole and negative corona present on the terminal iodine atoms [34,35,36]. Accordingly, the crystal packing of [Au(Me_2_pipdt)I_2_]I_3_ shows the terminal I(3) atom of I_3_^−^ anion interacting with both I(1) and I(2) in a slightly asymmetric bifurcated interaction. The distances between I(3) and I(1) or I(2), approximately 3.7 Å, are significantly shorter than the sum of the Van der Waals radii of the iodine atoms (3.96 Å). The Au-I(1)/I(2)-I(3) angles are greater than 165° and in agreement with a σ-hole on I(1) and I(2) interacting with a negative corona on I(3) (Figure S1). On the opposite, the terminal I(5) interacts with the gold atom of a symmetry related molecule by virtue of its negative corona, Figure S2.

The crystal structure of the d^8^-metal [Pd(Me_2_pipdt)_2_](I)_2_ (**2a**) is shown in Figure 2 and the corresponding bond distances and angles are presented in Table 1. The asymmetric unit comprises half-complex cation and an iodide anion, whereas the overall molecular structure is represented by a square–planar cation and two iodide anions. The spherical charge distribution on I^−^, in contrast to the non-isotropic charge distribution on I_3_^−^, implies that I^−^ is mainly involved in weak CH···I hydrogen bonds (HBs) (Figure 2) [37]. Additional weak CH···S HBs between symmetry related molecules contribute to the crystal packing, Figure S3.

**Figure 6:**
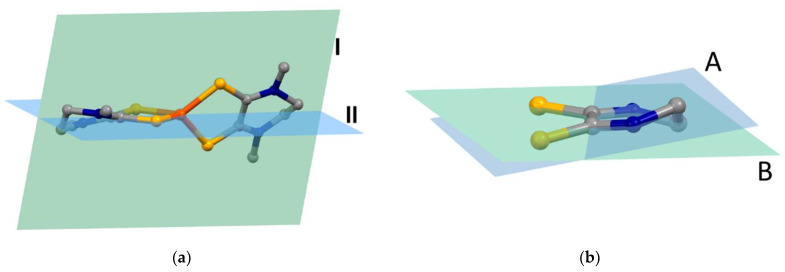
Depiction of the molecular planes used to calculate the dihedral angle between the S,S-chelate ligands (**a**) and between the thioamido groups (**b**). See also Tables S2 and S3.

The three Cu(I) complexes are characterized by a distorted tetrahedral geometry (Figure 3, Figure 4 and Figure 5). In order to better describe the structural features, it is useful to employ the τ_4_ and τ_4′_ geometry indices index, which have different values according to a specific ideal geometry: (i) τ_4_ = τ_4′_ = 0 for square–planar systems; (ii) τ_4_ = τ_4′_ = 1 for a tetrahedral geometry; and iii) τ_4_ = 0.43 and τ_4′_ = 0.24 for a seesaw geometry [38,39]. The values of τ_4_ and τ_4′_ are greater than 0.6 for all the copper complexes (Table 2) and the distortion from the ideal tetrahedral geometry that may be adopted by Cu(I), can be ascribed to the presence of bidentate ligands that form five membered chelate rings, with bite angles close to 90° (Table 3). Moreover, [Cu(Me_2_dazdt)_2_]I_3_ exhibits the greatest τ_4_ and τ_4′_ values of the three complexes, hence it appears to adopt a more regular tetrahedral geometry. The reason for this behavior can be explained by considering that the two thioamido groups of the Me_2_dazdt ligand present a dihedral angle of more than 50°, whereas the same angle for the Me_2_pipdt ligand is approximately around 20° (range 9.1–27.8°), as pointed out by Tables S2 and S3 and Figure 6.

This implies that the intra-molecular sulfur–sulfur distance for Me_2_dazdt is approximately 0.15 Å greater than that found in the Me_2_pipdt ligand. Consistently, the bite angle is significantly greater in [Cu(Me_2_dazdt)_2_]I_3_ than in the other two Cu(I) complexes. Obviously, the presence of the seven membered ring in the Me_2_dazdt molecular framework, confers to this ligand a higher degree of conformational flexibility and the ligand is presumably able to adapt to the stereo-electronic requirement of the metal ion. Additionally, for the Cu(I) complexes, the crystal packing features heavily depend on the counter-anion. Specifically, the tetrahedral shape of BF_4_^−^ allows for interactions with the surrounding molecules distributed over a wider 3D surface than the linear I_3_^−^ anion. Another notable difference between BF_4_^−^ and I_3_^−^ is the higher polarizability of the iodine atoms, which can influence the non-uniform distribution of the electron density over the molecular surface (vide supra). The packing of [Cu(Me_2_pipdt)_2_]BF_4_ is mainly dominated by CH···F interactions occurring between the anions and the aliphatic CH_2_ or CH_3_ groups of the ligand. On the other hand, in [Cu(Me_2_dazdt)_2_]I_3_ and [Cu(Me_2_pipdt)_2_]I_3_, the packing is also influenced by the features of the I_3_^−^ anion, which can give rise to halogen–halogen and weak hydrogen bond interactions. More specifically, in [Cu(Me_2_pipdt)_2_]I_3_ the central iodide atom of I_3_^−^ interacts with the CH_2_ groups of the ethylene bridge. The terminal atom I(3) interacts also with a CH_2_ group, but the H···I(3)-I(2) angle is approximately 155° and far from linear, since this interaction most-probably involves the diffuse negative corona on I(3). According to the geometry of the interaction between two symmetry-related I_3_^−^ anions, there seems to be a very weak type I halogen interaction [40]. Indeed, the angles I(2)-I(1)···I(3) and I(2)-I(3)···I(1) are 130 and 128°, respectively, but the I(1)···I(3) distance of 4.09 Å is slightly greater than the sum of the iodine Van der Waals radii. The crystal packing of [Cu(Me_2_dazdt)_2_]I_3_ shows the presence of a halogen bond (type II halogen contact) between an I_3_^−^ that, with the terminal iodine atom I(16), interacts with the central iodine atom I(25) of a second I_3_^−^. As pointed out before, according to the electron density distribution on I_3_^−^, the terminal iodine is characterized by the presence of a σ-hole (a less negative region than the rest of the molecule, since it possesses a net negative charge) [35] and it can give rise to a halogen bond. Consistently, the I(16)···I(25) distance of 3.82 Å is significantly smaller than the Van der Waals radii sum of the two iodines. Additional interactions between the two cationic molecules of the asymmetric unit involve the CH_2_ and CH_3_ and the electron rich sulfur atoms, Figures S4 and S5.

A last structural aspect is related to the analysis of bond distances within the thioamido groups. Indeed, given the nature of these ligands, different resonance forms related to the thione or to the thiolate limit structures, may be written. Restricting the analysis to the Cu(I) complexes, the C=S distances are in the narrow range of 1.70–1.67 Å, with the mean value around 1.68 Å. The C–N distances are in the 1.33–1.29 Å (mean value 1.32 Å), hence the NCS system can be mostly described with a thione-like structure. The bond distance between the carbon atoms linking the thioamido groups is usually greater than 1.50 Å, pointing to a single bond character and with no electron delocalization between the two thioamido moieties within a ligand.

### 2.3. Vibrational and Electronic Spectral Features

Vibrational spectroscopic data are consistent with structural findings (Table S4 and Figures S6–S9). Specifically, a shift to higher frequency of the ν(CN) vibration is observed for **1**–**5** (**1** > **2** > **3 ~ 4 ~ 5**) due to the increase of the CN double bond character of the thioamide moiety on sulfur coordination. In the region typical of interiodine vibrations [41], the Raman spectra of **2**, **3** and **5** show the presence of peaks typical of I_3_^−^ at approximately 110 cm^−1^ (vs) and 140 cm^−1^ (m). These peaks are assigned respectively to the Raman-active symmetrical and to the antisymmetrical stretching which becomes Raman-active for asymmetrical I_3_^−^ units. Differently, in the case of **1**, no strong peak near 110 cm^−1^ is observed in the spectrum. This reflects the peculiar features of this triiodide, where one of the terminal atoms interacts with both iodine atoms coordinated to the metal, while the other interacts with gold of a symmetry related molecule. These interactions, which may be limited to the solid state, seem partially active in solution, where absorbances of the typical peaks of triiodides at 290 and 360 nm are approximately one half lower than predictable for **1**, differently to what was observed for **2**, **3** and **5**. This may suggest that triiodide undergoes equilibrium reactions where iodine and coordinated iodides likely compete to interact with iodide. As far as the electronic spectra of [Pd(Me_2_pipdt)_2_]I_6_ (**2**) are concerned, in addition to the typical absorptions of triiodides at 290 and 360 nm, two peaks at 420(sh) nm and 450 nm appear in the visible region. These peaks have been formerly assigned to transitions between (HOMO-1)-LUMO and HOMO-LUMO frontier orbitals [42], determined by approximate extended Hückel calculations (CACAO software) [43]. In these π-type orbitals, the contribution of the metal to the empty orbitals is negligible, while in the populated ones it is high (highest for Pd in the Ni-triad) and affects their energy [44]. DFT calculations provide further support to these findings (see DFT Calculations section).

In the electronic spectra of [Cu(Me_2_pipdt)_2_]I_3_ (**3**), in addition to the triiodide related peaks, a peak appears at 530 nm. This peak can be assigned to a metal-to-ligand charge-transfer transition, according to recent studies on this class of complexes [28].

### 2.4. DFT Calculations

In order to further investigate the electronic structures of the cationic part of salts **1**–**3** and **5** (**1′**–**3′** and **5′**), density functional theory (DFT) calculations were performed using B3LYP as functional. The gas phase optimized geometries reported in Figure S10 are in reasonably good agreement with those obtained by X-ray measurements. A comparison between experimental and calculated bond distances and angles is reported in Tables S5 and S6. In the case of **1**, calculated Au-I and C-S bond distances are in close agreement with the found ones, while the Au-S or M-S ones are slightly overestimated, as typically found with DFT functionals [45,46]. The calculated bite angles are a few degrees smaller than those measured.

**Figure 7:**
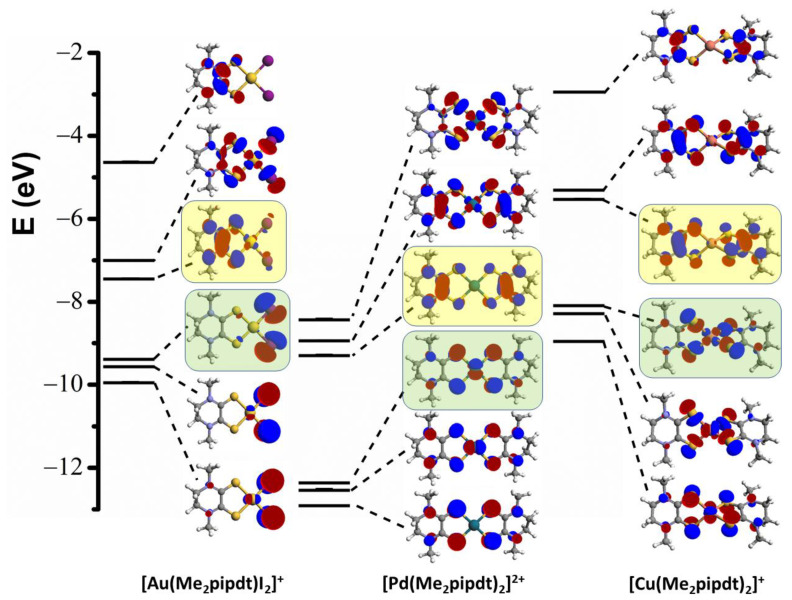
DFT calculated molecular orbitals and orbital diagram for cationic complexes **1′**, **2′** and **3′**. Starting from the upper part, the figure depicts all the MO ranging from the LUMO+2 to the HOMO-2; for each complex, HOMO and LUMO are highlighted in pale green and yellow colors, respectively (isovalue = 0.04).

In Figure 7, shapes and energies diagrams of molecular orbitals of the complexes **1′**–**3′** are reported.

The nature and the energy of the frontier orbitals is markedly different in the three cases. In complex **1′**, the highest occupied molecular orbital (HOMO) is almost completely composed of *p* iodide orbitals; the same depiction can be also used for HOMO-1 and HOMO-2 MOs which, in addition, present small contributions from gold 5d orbitals, due to its high effective nuclear charge which stabilizes the *d*-metal orbitals with respect to the ligand orbitals, preventing significant mixing between them. The lowest unoccupied molecular orbital (LUMO) is predominantly pipdt-ligand-based with small gold and iodide contribution in *π*-antibonding combination, while the LUMO+1 is a combination of pipdt-ligand-based and iodide with *d*-Au orbitals, in σ-antibonding interaction. Complex **2′** presents frontier orbitals (FOs) with lower energies compared to the corresponding ones of **1′**. This lowering is relatable to the Pd(II) lower nuclear effective charge which allows a significant metal contribution to the π-electron system of the two Me_2_pipdt ligands. The related sequence of frontier *π*-MOs can be taken as typical of dicationic dithiolenic systems. The HOMO is formed by the out-of-plane interactions between a *d_π_* Pd orbital and a π-ligand orbital with mainly sulfur *p* character. The lowest unoccupied molecular orbitals are mainly formed by ligands orbitals with C–C *π*, C–S π*, and C–N π* character, in the out-of-phase (LUMO) and the in-phase (LUMO+1) combinations between them, with negligible contribution from the metal. Electrochemical data on salts of [Pd(R_2_pipdt)_2_]^2+^ [24], showing four reversible redox steps relatable to a stepwise reduction from the dication to the dianion, further support that **2′** can be taken as a rare example of a dicationic dithiolene. In the copper(I) complex **3′**, the tetrahedral geometry allows for a bonding interaction between the sulfur *p* and metal *d* orbitals. These results are in agreement with those previously reported by P. Basu et al. [27,28], on a study of Cu(I) (see Figure S11) and Cu(II) complexes with the same or similar ligands (Me_2_pipdt and *^i^*Pr_2_pipdt). In addition to crystals of [Cu(*^i^*Pr_2_pipdt)_2_][PF_6_] and [Cu(Me_2_pipdt)_2_][PF_6_], these authors succeed in isolating crystals of a Cu(II) derivative: [Cu(*^i^*Pr_2_pipdt)_2_][BF_4_]_2_. In agreement with our findings, but with longer times, the spontaneous reduction of Cu(II) to Cu(I) complexes in solution, even under inert atmosphere conditions, was observed. As predictable the Cu(I) complexes have a distorted tetrahedral geometry, whereas the Cu(II) complex exhibits a square–planar geometry. Computational studies on [Cu(*^i^*Pr_2_pipdt)_2_][PF_6_]_n_, *n* = 1 and 2, have also been performed and the sequence of molecular orbitals and orbital diagram for **3′**, shown in Figure 7, is in agreement with that reported for [Cu(*^i^*Pr_2_pipdt)_2_]^+^. Notably, the sequence of the frontiers orbitals suggests that, on reduction, the change from the square–planar Cu(II) paramagnetic complex [Cu(*^i^*Pr_2_pipdt)_2_]^2+^ to the tetrahedral Cu(I) derivative [Cu(*^i^*Pr_2_pipdt)_2_]^+^, turns the *σ*-antibonding interaction in a partial-bonding overlap between the sulphur *p* and metal *d* orbitals, as also observed in **3** and **5**. As discussed in the Molecular Structures section, the distortion from the ideal tetrahedral geometry observed in **3**, **4** and [Cu(*^i^*Pr_2_pipdt)_2_]^+^ can be ascribed to the constrains of R_2_pipdt ligands that form six membered chelate rings. Instead the seven membered ring in Me_2_dazdt confers to this ligand a higher degree of conformational flexibility, allowing [Cu(Me_2_dazdt)_2_]^+^ to adopt a more regular tetrahedral geometry with more effective metal–sulfur interactions. A comparison between the MOs of complexes **3′** and **5′** (see Figure S12) shows similar electronic structures, although there is a difference in the order of MOs with HOMO and HOMO-1 swapped.

Cyclic voltammetry results on [Cu(R_2_pipdt)_2_]^+^ derivatives, previously reported [27,28], show the presence of a reversible Cu(II/I) couple at ca. 100 mV, but no reversible ligand based peaks. Thus, the loss of planarity on metal reduction seems to further support that in the [Cu(R_2_pipdt)_2_]^2+^/[Cu(R_2_pipdt)_2_]^+^ system the dithiolenic nature of complexes cannot be invoked, differently to the case of [Pd(R_2_pipdt)_2_]^2+^ (**2′**).

### 2.5. Recovery of Metals from the Leachate

As described above, Me_2_pipdt/I_2_ mixtures have been demonstrated to be effective agents for gold, palladium and copper dissolution, working through a one-pot reaction in very mild conditions.

In the specific case, the triad of metals here described is typically contained in the metal mixtures deriving from waste electric and electronic equipment like printed circuit boards where copper represents the most abundant non-ferrous metal (80%) and palladium and gold are present in low but significant amounts (0.01–0.1%). For practical purposes, an unselective leaching based on the use of the effective but relatively expensive Me_2_pipdt/I_2_ mixture does not seem convenient since it would also require further separation and concentration steps for achieving a selective metal recovery. Instead, a stepwise process which includes a preliminary selective leaching of copper (and other base metals still present in the mixture) by a cheaper more conventional reagent, such as NH_3_ in an oxidizing environment, or others [47,48,49], may add effectiveness and sustainability to the process, which still remains cost-effective for Au and Pd. Accordingly, experiments to recover the metal, Au or Pd, from the corresponding leachates were performed. Specifically, conventional cementation experiments were performed by dissolving **1** and **2** in a small amount of CH_3_CN and reacting with an excess of Mg flakes under stirring at room temperature. Despite a reaction seeming to occur in both cases, only the reaction of **1**, were gold metal and MgI_2_ collected from the bottom of the flask while Me_2_pipdt was recovered from solution at the end of the process, to be fully recycled as detailed in the Section 3.2.

On the other hand, the above described treatment with Mg does not work with **2**, as previously observed for the corresponding [Pd(Me_2_dazdt)_2_]I_6_ salt [29], to recover palladium metal and the ligand. This seems to be associated with the dithiolenic nature of these complexes, which is preserved even following reduction. Indeed, cyclic voltammetry of these compounds shows the presence of four reversible reduction waves corresponding to the reduction of the complex starting from 2+ and lasting to 2- [42], which can be related to the stepwise electron addition to LUMO and LUMO+1, in agreement with DFT calculation findings (see Figure 7). The different behavior of gold and palladium complexes under reducing conditions, may add selectivity to a recovery process where both metals are leached together. Indeed, gold(III) can be selectively reduced to gold(0) while **2** remains in solution.

Despite the fact that an alternative thermal degradation of **2** to recover palladium metal can be performed, it seems more appealing in terms both of economic and environmental aspects, to investigate the use of compound **2** in high valuable applicative fields such as homogeneous catalysis, where this class of compounds has already demonstrated their promising properties [50].

## 3. Materials and Methods

Reagents and solvents were purchased from Sigma-Aldrich and used without further purification. Metal powders were purchased as follows: Au (Alfa Aesar, 0.5–0.8 μm, 99.99%); Pd (Alfa Aesar, −200 mesh, 99.95%) and Cu (Carlo Erba, ≥98%).

Complexes were prepared in several solvents (e.g., THF, CH_3_CN) by a twofold approach: (1) using a solution of the ligand (Me_2_pipdt or Me_2_dazdt) in the presence of the appropriate amount of I_2_; or (2) starting from a solution of the solid-state isolated charge-transfer compound obtained by reacting the ligand with iodine (namely [Me_2_pipdtH]I_3_ and Me_2_dazdt·2I_2_, respectively), obtaining almost the same results. Approach (2) using THF as solvent will be detailed for brevity reasons.

CHN-analysis was performed on a Carlo Erba CHNS elemental analyzer model EA1108. MIR spectra (4000–200 cm^−1^) were recorded on the crude sample with a Jasco FT-IR6300A spectrometer equipped with a ATR PRO ONE (diamond crystal) and, for compound **1**, on KBr pellets with a Perkin Elmer model 983 spectrometer in the 4000–400 cm^−1^ spectral range. FIR spectra (300–50 cm^−1^) were recorded on polyethylene pellets with a Bruker IFS55 FT-spectrometer. FT-Raman spectra were recorded on solid sample in a capillary tube, resolution ±4 cm^−1^, power 50 mW, Bruker model RFS100/S FT-spectrometer, operating with an excitation frequency of 1064 nm, Nd:YAG laser, and an Indium–Gallium–Arsenide detector. Electronic spectra were recorded by a Varian Cary 5 Spectrophotometer, on 1.0 × 10^−4^ M CH_3_CN solutions of the compounds contained in a 0.1 cm silica cell at room temperature. ^1^H and ^13^C-deptq135 NMR experiments and 2D ^1^H-^13^C HSQC were performed on a Brüker Avance 400 MHz instruments at 298 K. Chemical shifts are quoted in ppm relative to tetramethylsilane, using the solvent residual peak of DMSO-*d*_6_ (δH 2.50, δC 39.51) CD_3_CN (δH 1.94, δC 1.39 and 118.69) as a reference standard. The ^13^C- deptq135 spectra were phased according to: Cquat/CH_2_ up and CH/CH_3_ down.

### 3.1. Synthesis of Reagents and Products

[Me_2_pipdtH]I_3_ and [Me_2_dazdt·2I_2_] were prepared according to refs. [16,24], respectively.

*[AuI_2_(Me_2_pipdt)]I_3_* (**1**). Au powder (17.7 mg, 0.0900 mmol) was reacted with a THF solution of [Me_2_pipdtH]I_3_ (0.100 g, 0.180 mmol in 0.100 L) under stirring at room temperature. The solution darkened from red-brown to brown and a fast precipitation occurred during the formation of the complex. In about 1 h the metal powder disappeared. Dark-brown well-shaped crystals of **3**, suitable for X-ray studies, were precipitated with a 70% yield (63.3 mg, 0.063 mmol), from THF/Et_2_O, and were washed with Et_2_O. Analysis found: C% 8.34; H% 1.05; N% 3.01; S% 6.81; Calcd. for AuC_6_H_10_N_2_S_2_I_5_ (1005.758): C% 8.20; H% 1.15; N% 3.19; S% 7.30. MIR (on KBr pellets, cm^−1^): 2970vw, 2930vw, 1552vs(νCN), 1405w, 1385m, 1365s, 1285mw, 1265w, 1140m, 1090w, 1015w, 535w, 420vw. Raman (cm^−1^): 2968vw, 2902vw, 1555mw, 1431vw, 1402vw, 1364m, 1283vw, 1140vw, 530vw, 428vw, 392m, 325vw, 197ms, 162s, 150vs, 139-sh, 101ms, 76ms. UV-Vis (in CH_3_CN) [λ = nm (ε = dm^3^ mol^−1^ cm^−1^)]: 217(24,000), 234-sh, 295(34,000), 352(10,000), 503(2500). ^1^H-NMR (400 MHz, CD_3_CN): δ, 3.71 (s, 3H, CH_3_), 4.15 (s, 2H, CH_2_). ^13^C-NMR (100 MHz, CD_3_CN): δ, 47.00 (CH_3_), 52.64 (CH_2_) ppm.

*[Pd(Me_2_pipdt)_2_](I_3_)_2_* (**2**). Pd powder (9.59 mg, 0.0900 mmol) was reacted with a THF solution of [Me_2_pipdtH]I_3_ (0.100 g, 0.180 mmol in 0.100 L) under stirring at room temperature. The solution darkened rapidly from red-orange to dark-brown with the formation of the complex. After about 2 h the palladium powder disappeared. Red-brown crystals of **1** (52.7 mg, 0.045 mmol) were obtained from THF/Et_2_O and washed with Et_2_O (50% yield). Analysis found: C% 12.09; H% 1.57; N% 4.54; S% 10.35; Calcd. for PdC_12_H_20_N_4_S_4_I_6_ (1168.67): C% 11.85; H% 1.66; N% 4.61; S% 10.54. MIR (on KBr pellets, cm^−1^): 2970w, 2902w, 1538vs (νCN), 1426m, 1393s, 1359vs, 1268s, 1258s, 1205w, 1185vw, 1141m, 1135w, 1115m, 890m, 813m, 665m, 599m, 539s, 454vs, 430m. Raman (cm^−1^): 2959w, 2910w, 2845vw, 1549mw, 1444w, 1396w, 1367m, 1291vw, 1265mw, 1149w, 544w, 430vw, 388w-br, 147s, 133s, 109vs. UV-Vis (in CH_3_CN) [λ = nm (ε = dm^3^ mol^−1^ cm^−1^)]: 290 (117,000), 361(48,000) 2 I_3_^−^, 425-sh, 450(18,000). Well-shaped crystals, suitable for single-crystal X-Ray diffraction measurements, were obtained after several recrystallization attempts by dissolution/crystallization processes in THF/Et_2_O, obtaining the corresponding [Pd(Me_2_pipdt)_2_]I_2_ complex. ^1^H-NMR (400 MHz, DMSO-*d*_6_): δ, 3.66 (s, 3H, CH_3_), 4.19 (s, 2H, CH_2_); ^13^C-NMR (100 MHz, DMSO-*d*_6_): δ, 44.86 (CH_3_), 50.92 (CH_2_), 181.69 (C=S) ppm.

*[Cu(Me_2_pipdt)_2_]I_3_* (**3**). Cu powder (5.73 mg, 0.0900 mmol) was reacted with a THF solution of [Me_2_pipdtH]I_3_ (0.100 g, 0.180 mmol in 0.100 L) under stirring at room temperature. The solution darkened readily from red-brown to dark-red. Purple well-shaped crystals of **2**, suitable for X-ray studies, were obtained in an almost quantitative yield (70.9 mg, 0.0996 mmol) from THF/Et_2_O, and washed with Et_2_O. Analysis found: C% 18.36; H% 2.57; N% 7.03; S% 16.49; Calcd. for CuC_12_H_20_N_4_S_4_I_3_ (792.83): C% 18.18; H% 2.54; N% 7.07; S% 16.18. MIR (on KBr pellets, cm^−1^): 2960vw, 2912w, 2854vw, 1512vs (νCN), 1462w-br, 1425w, 1397s, 1348vs, 1281mw, 1262vs, 1197mw, 1158w, 1129m, 1096m, 1030w, 1015w, 897s, 815w, 677m, 594w, 541s, 467s, 412w. FIR (on polythene pellets, cm^−1^): 225w, 200w, 140vs, 117mw, 82mw, 73-sh. Raman: the sample showed fluorescence. However, intense peaks at 130s and 113vs cm^−1^ were present. UV-Vis (in CH_3_CN) [λ = nm (ε = dm^3^ mol^−1^ cm^−1^)]: 225(28,000), 292(62,000), 362(22,000) I_3_^−^, 530(11,000). ^1^H-NMR (400 MHz, DMSO-*d*_6_): δ, 3.59 (s, 3H, CH_3_), 3.98 (s, 2H, CH_2_); ^13^C-NMR (100 MHz, DMSO-*d*_6_): δ, 47.59 (CH_3_), 49.56 (CH_2_), 182.01 (C=S), ppm.

*[Cu(Me_2_pipdt)_2_]BF_4_* (**4**). 0.2 g of Me_2_pipdt and 0.08 g of CuCl_2_ were dissolved in 100 mL of THF and reacted under reflux in Ar atmosphere and magnetic bar stirring with an excess of NH_4_BF_4_. In these conditions, the solution turned from light to dark green, then to purple when the reaction went off in around 1 h. Crystalline product **4** was recovered by the cold solution after addition of EtOH (in 1:1 ratio in volume with THF) and slow evaporation of THF by heating the mixture under stirring, and fully characterized. Analysis found: C% 29.38; H% 4.14; N% 11.50; S% 25.42; Calcd. for CuC_12_H_20_N_4_S_4_BF_4_ (498.905): C% 28.89; H% 4.04; N% 11.23; S% 25.71. MIR (ATR, cm^−1^): 3008vw, 2981vw, 2936w, 2909w, 1511vs (νCN), 1447w, 1432w, 1406m, 1391-sh, 1347vs, 1283m, 1266m, 1204w, 1157vw, 1126w, 1086m, 1027vs, 898m, 876-sh, 812w, 686m, 597w, 541s, 519m, 445w. ^1^H-NMR (400 MHz, DMSO-*d*_6_): δ, 3.59 (s, 3H, CH_3_), 3.98 (s, 2H, CH_2_); ^13^C-NMR (100 MHz, DMSO-*d*_6_): δ, 47.12 (CH_3_), 49.09 (CH_2_), 181.54 (C=S), ppm.

*[Cu(Me_2_dazdt)_2_]I_3_* (**5**). Cu powder (4.57 mg, 0.0720 mmol) was reacted with a THF solution of Me_2_dazdt·2I_2_ (0.100 g, 0.144 mmol in 0.100 L) under stirring at room temperature. The solution darkened rapidly from red-orange to red-brown with the formation of the complex. Red-brown needle crystals of **5** (59.0 mg, 0.0719 mmol) were crystallized, in an almost quantitative yield, from THF/Et_2_O, and were washed with Et_2_O. Analysis found: C% 20.60; H% 3.03; N% 6.62; S% 15.38; Calcd. For CuC_14_H_24_N_4_S_4_I_3_ (820.884): C% 20.48; H% 2.95; N% 6.82; S% 15.63. MIR (on KBr pellets, cm^−1^): 2960-sh, 2922w, 1513vs (νCN), 1451mw, 1435w, 1394s, 1356m, 1331w, 1279s, 1254m, 1183w, 1102m, 1074w, 1025w, 967w, 887vw, 827m, 745w, 691vw, 612m, 582w, 532m, 449vw. FIR (on polythene pellets, cm^−1^): 140s, 106m. Raman (cm^−1^): 2954vw, 2929w, 2892vw, 1530w, 1457vw, 1399mw, 1358vw, 1258mw, 1211w, 1122vw, 891vw, 619vw, 444mw, 409mw, 292mw, 164s, 151m, 135w, 111vs, 82mw-sh, 75mw-sh. UV-Vis (in CH_3_CN) [λ = nm (ε = dm^3^ mol^−1^ cm^−1^)]: 237(39,000), 293(70,000), 363(35,000) I_3_^−^. ^1^H-NMR (400 MHz, DMSO-*d*_6_): δ, 2.20 (m, 2H, CH_2_), 3.46 (br s, 8H, CH_2_ and CH_3_), 3.75 (br m, 2H, CH_2_); ^13^C-NMR (100 MHz, DMSO-*d*_6_): δ, 27.27 (CH_2_), 42.26 (CH_3_), 52.10 (CH_2_), ppm.

### 3.2. Procedure for Gold and Reagents Recovery from Complex ***1***

28 mg of complex **1** were dissolved in 40mL of CH_3_CN at room temperature. To this solution an excess of shiny Mg flakes was added and stirred. After few minutes, the solution color turned from orange-brown to yellow and Mg flakes darkened for a dark powder deposition on the surface. This reaction was also accompanied by a precipitation of whitish needle crystals supposed to be MgI_2_. Mg flakes were separated by the solution (containing the white precipitate) and dissolved by diluted HCl, leaving a black powder unreacted, supposed gold metal. The black powder was, then, treated for gold identification and quantification by dissolution with few mL of aqua regia (forming a yellow solution), HNO_3_ removal by heating under the fume hood and gold reduction to gold(0) by addition of Na_2_SO_3_ (up to persistent smell of SO_3_), obtaining gold powder in a >90% yield. The yellow solution containing the whitish precipitated was dried, then the solid residue washed by water for extracting soluble MgI_2_. The solid residue was redissolved in CH_3_CN and characterized by UV-Vis spectrophotometry which showed the typical spectrum of the free ligand. MgI_2_ was identified as follows: the iodide by quantitative precipitation of AgI by addition of an excess of AgNO_3_ into the water solution; and the Mg^2+^ through elemental analysis by Atomic Absorption Spectroscopy. In terms of complete recycling of the reagents for applicative purposes, iodine can be recycled by the I^−^ water solution by adding 30% H_2_O_2_, heating under stirring and I_2_ vapors frosting to crystalline I_2_ on the surface of a cold finger.

### 3.3. X-ray Measurements

Single crystal Data for complexes **1**, **2a**, **3**, **4**, and **5**, were collected at 220K with a Bruker D8 diffractometer equipped with a Photon II detector, using a MoKα microfocus radiation source (λ = 0.71073). The intensity data were integrated from several series of exposure frames covering the sphere of reciprocal space. Data reductions were performed with APEX3 [51] or with CrysAlysPro [52]. Absorption corrections were applied using the program SADABS [53]. The structures were solved with the program SHELXT [54]. Fourier analysis and refinement were performed by the full-matrix least-squares methods based on F^2^ using SHELXL-2014 [55], using Olex2 [56]. All the non-H atoms were refined with anisotropic displacement parameters. In [Cu(Me_2_pipdt)_2_]BF_4_ both of the BF_4_^−^ anions of the asymmetric unit were modelled over two distinct sites (with 0.83/0.17 and 0.86/0.14 site occupancy factors, respectively). The crystals of [Cu(Me_2_dazdt)_2_]I_3_ were non-merohedrally twinned, and the best data set could be obtained by using only one major crystalline domain and a narrow integration box to minimize reflection overlaps. CCDC 2084168-2084172 contains the Supplementary crystallographic data for this paper. These data can be obtained free of charge via http://www.ccdc.cam.ac.uk/conts/retrieving.html (or from the CCDC, 12 Union Road, Cambridge CB2 1EZ, UK; Fax: +44 1223 336033; E-mail: deposit@ccdc.cam.ac.uk).

### 3.4. DFT Calculations

Ground-state electronic structure calculations of [AuI_2_(Me_2_pipdt)]^+^ (**1′**), [Pd(Me_2_pipdt)_2_]^2+^ (**2′**), [Cu(Me_2_pipdt)_2_]^+^ (**3′**) and [Cu(Me_2_dazdt)_2_]^+^ (**5′**) were performed at DFT [57] level employing the GAUSSIAN 16 [58] software package. The Becke three-parameters exchange functional with Lee–Yang–Parr correlation functional (B3LYP) [59,60] was used. The ground state geometries were obtained in the gas phase by full geometry optimization without any symmetry constraints. The basis sets employed were 6-31+G** [61] (for C, H, N and S), LANL2DZ [62] (Cu) and Def2TZVPP [63] (Au, Pd and I) with pseudopotentials on Pd, Au and I atoms. All structures were input starting from the crystallographic data. The optimized molecular structures and the orbital isosurfaces were visualized using ArgusLab 4.0 [64].

## 4. Conclusions

In conclusion, the reported reactions show once more the combined action of DTO–diiodine mixture represents a valuable method for leaching noble metals in a surprisingly mild and effective way. The obtained products may be used to provide elemental metal [47,48,49], as here demonstrated for gold, or also as valuable candidates for applications in catalysis [50]. Furthermore, the structure and bonding description of compounds **1**–**5** sheds light on the role of the DTO-ligands in favoring the oxidation state and coordination of these metals. The found structure–property relationship may provide a useful contribution to studies on related applicative fields spanning from biochemistry to catalysis [50,65]. Focusing on the applicative purposes of these smart reactants, due to the cost related to their synthesis and precursors, their use at the practical level should be restricted to cases of selective leaching of limited amounts of these high value metals and/or when the organic ligand can be recycled besides the metal. Otherwise, the cost of the ligands would make the method uneconomic for application purposes at a large scale. Instead, high added value is ensured when the leaching products are suitable for applications as they are, avoiding the recovery process.

## Figures and Tables

**Table 1:** Selection of bond distances (Å) and angles (°) for compounds [Au(Me_2_pipdt)I_2_]I_3_ (**1**) and [Pd(Me_2_pipdt)_2_](I)_2_ (**2a**).

[Au(Me_2_pipdt)I_2_]I_3_ (1)		[Pd(Me_2_pipdt)_2_](I)_2_ (2a)	
Au-S(1)	2.316(2)	Pd-S(1)	2.2952(5)
Au-S(2)	2.319(2)	Pd-S(2)	2.2826(5)
Au-I(1)	2.6113(4)	C(1)-S(1)	1.700(2)
Au-I(2)	2.6139(5)	S(2)-C(2)	1.701(2)
C(1)-S(1)	1.694(5)	C(1)-N(1)	1.313(2)
C(2)-S(2)	1.699(6)	C(2)-N(2)	1.307(2)
C(1)-N(1)	1.311(7)	C(1)-C(2)	1.500(3)
C(2)-N(2)	1.319(7)		
C(1)-C(2)	1.493(7)		
I(3)-I(4)	3.0419(5)		
I(4)-I(5)	2.8293(6)		
S(1)-Au-S(2)	89.97(5)	S(1)-Pd-S(2)	88.58(2)

**Table 2:** Values of τ_4_ and τ_4_′ indices with coordination angles β and α (β > α).

Complex	β	α	τ_4_	τ_4_′
[Au(Me_2_pipdt)I_2_]I_3_	177.72	177.52	0.03	0.03
[Pd(Me_2_pipdt)_2_](I)_2_	180	180	0.00	0.00
[Cu(Me_2_pipdt)_2_]I_3_	133.81	132.67	0.66	0.66
[Cu(Me_2_pipdt)_2_]BF_4_ *	125.39	116.23	0.84	0.81
	126.9	125.09	0.77	0.76
[Cu(Me_2_dazdt)_2_]I_3_ *	113.8	111.11	0.96	0.95
	124.23	122.8	0.80	0.80

* two complex molecules are present in the asymmetric unit.

**Table 3:** Selection of bond distances (Å) and angles (°) for compounds [Cu(Me_2_pipdt)_2_]I_3_ (**3**), [Cu(Me_2_pipdt)_2_]BF_4_ (**4**) and [Cu(Me_2_dazdt)_2_]I_3_ (**5**).

[Cu(Me_2_pipdt)_2_]I_3_(3)		[Cu(Me_2_pipdt)_2_]BF_4_(4)		[Cu(Me_2_dazdt)_2_]I_3_(5)	
Cu-S(11)	2.272(2)	Cu(1)-S(11)	2.260(1)	Cu(1)-S(11)	2.342(4)
Cu-S(21)	2.295(2)	Cu(1)-S(21)	2.300(1)	Cu(1)-S(21)	2.344(4)
C(11)-S(11)	1.689(5)	C(11)-S(11)	1.683(4)	C(11)-S(11)	1.69(1)
C(21)-S(21)	1.679(6)	C(21)-S(21)	1.682(3)	C(21)-S(21)	1.69(2)
C(11)-N(11)	1.323(7)	C(11)-N(11)	1.320(4)	C(11)-N(11)	1.29(2)
C(21)-N(21)	1.322(7)	C(21)-N(21)	1.319(4)	C(21)-N(21)	1.34(2)
C(11)-C(21)	1.515(8)	C(11)-C(21)	1.520(5)	C(11)-C(21)	1.49(2)
Cu-S(12)	2.276(2)	Cu(1)-S(12)	2.274(1)	Cu(1)-S(12)	2.309(4)
Cu-S(22)	2.283(2)	Cu(1)-S(22)	2.302(1)	Cu(1)-S(22)	2.338(4)
C(12)-S(12)	1.679(5)	C(12)-S(12)	1.681(3)	C(12)-S(12)	1.69(1)
C(22)-S(22)	1.670(5)	C(22)-S(22)	1.687(3)	C(22)-S(22)	1.69(1)
C(12)-N(12)	1.313(7)	C(12)-N(12)	1.326(4)	C(12)-N(12)	1.29(2)
C(22)-N(22)	1.318(7)	C(22)-N(22)	1.312(4)	C(22)-N(22)	1.31(2)
C(12)-C(22)	1.523(8)	C(12)-C(22)	1.520(5)	C(12)-C(22)	1.52(2)
S(11)-Cu-S(21)	91.71(6)	S(11)-Cu(1)-S(21)	91.16(4)	S(11)-Cu(1)-S(21)	93.1(1)
S(12)-Cu-S(22)	91.24(5)	S(12)-Cu(1)-S(22)	90.99(4)	S(12)-Cu(1)-S(22)	93.6(1)
		Cu(2)-S(13)	2.279(1)	Cu(2)-S(13)	2.312(4)
		Cu(2)-S(23)	2.298(1)	Cu(2)-S(23)	2.335(4)
		C(13)-S(13)	1.682(4)	C(13)-S(13)	1.68(1)
		C(23)-S(23)	1.673(3)	C(23)-S(23)	1.70(1)
		C(13)-N(13)	1.320(4)	C(13)-N(13)	1.33(2)
		C(23)-N(23)	1.318(4)	C(23)-N(23)	1.32(2)
		C(13)-C(23)	1.518(5)	C(13)-C(23)	1.52(2)
		Cu(2)-S(14)	2.268(1)	Cu(2)-S(14)	2.301(4)
		Cu(2)-S(24)	2.277(1)	Cu(2)-S(24)	2.361(4)
		C(14)-S(14)	1.680(3)	C(14)-S(14)	1.67(1)
		C(24)-S(24)	1.678(3)	C(24)-S(24)	1.68(1)
		C(14)-N(14)	1.319(4)	C(14)-N(14)	1.33(2)
		C(24)-N(24)	1.320(4)	C(24)-N(24)	1.32(2)
		C(14)-C(24)	1.519(5)	C(14)-C(24)	1.51(2)
		S(13)-Cu(2)-S(23)	91.73(4)	S(13)-Cu(2)-S(23)	94.4(1)
		S(14)-Cu(2)-S(24)	92.20(4)	S(14)-Cu(2)-S(24)	92.9(1)

## Data Availability

Data are contained within the article or Supplementary Materials.

## Supplementary Materials

The following are available online, Table S1: Crystallographic data for compounds **1**–**5**; Tables S2 and S3: Dihedral angles for compounds **1**–**5**; Table S4: Vibrational peaks for **1**, **2**, **3**, **5**; Tables S5 and S6: Found vs. calculated bond distances and angles for **1′**, **2′**, **3′**, **5′**; Figure S1: Electrostatic potential map for **1′**; Figures S2-S5: Crystal packing of complexes **1**–**5**. Figures S6-S9: Vibrational spectroscopy characterization of **1**, **2**, **3**, **5**; Figure S10: Gas-phase DFT optimized geometries for **1′**, **2′**, **3′**, **5′**; Figure S11: DFT MO for **3′**; Figure S12: DFT MO for **3′** and **5′**; Figures S13–S23: ^1^H and ^13^C-NMR spectra for **1**–**5**.
