# Supplementary material for: Characterization and Structural Insights of the Reaction Products by Direct Leaching of the Noble Metals Au, Pd and Cu with N,N′-Dimethyl-piperazine-2,3-dithione/I2 Mixtures
Source: Molecules. 2021 Aug 4;26(16):4721. doi: 10.3390/molecules26164721 (PMC8400658; doi:10.3390/molecules26164721)
Supplement: Supplementary file 1 [file molecules-26-04721-s001.zip › molecules-1245880-supplementary.pdf]

Supplementary Material

for

Characterization and structural insights of the reaction products by direct leaching of the Noble Metals Au, Pd and Cu with N,N'-dimethyl-piperazine-2,3-dithione/I_2 mixtures.

Angela Serpe,* Luca Pilia, Davide Balestri, Luciano Marchiò,* Paola Deplano*

S1. X-Ray cristal data

Table S1. Summary of crystallographic data for compounds $[Au(Me_2pipdt)I_2]I_3$ (**1**), $[Pd(Me_2pipdt_2](I)_2$ (**2a**), $[Cu(Me_2pipdt)_2]I_3$ (**3**), $[Cu(Me_2pipdt)_2]BF_4$ (**4**) and $[Cu(Me_2dazdt)_2]I_3$ (**5**).

	**1**	**2a**	**3**	**4**	**5**
Empirical formula	$C_6H_{10}AuI_5N_2S_2$	$C_{12}H_{20}I_2N_4PdS_4$	$C_{12}H_{20}CuI_3N_4S_4$	$C_{12}H_{20}BCuF_4N_4S_4$	$C_{14}H_{24}CuI_3N_4S_4$
Formula weight	1005.75	708.76	792.80	498.91	820.85
Temperature/K	220.0	220.0	220.0	220.0	220
Crystal system	triclinic	monoclinic	triclinic	triclinic	monoclinic
Space group	P-1	$P2_1/n$	P-1	P-1	$P2_1/n$
a/Å	7.9419(2)	6.4232(2)	8.2504(4)	11.4721(9)	12.9755(6)
b/Å	10.3647(3)	13.5740(4)	8.9451(5)	12.9032(10)	30.1536(12)
c/Å	11.8639(3)	12.0555(3)	18.2307(10)	14.4528(11)	13.1801(6)
α/°	94.4790(10)	90	77.398(2)	89.393(3)	90
β/°	96.2720(10)	96.0560(10)	80.894(2)	87.028(3)	90.965(4)
γ/°	107.9910(10)	90	64.563(2)	66.124(3)	90
Volume/Å^3		1045.23(5)	1182.55(11)	1953.6(3)	5156.1(4)
Z	2	2	2	4	8
ρ_{calc}g/cm^3	3.643	2.252	2.226	1.696	2.115
μ/mm^{-1}	16.651	4.243	5.197	1.587	4.771
F(000)	872.0	672.0	744.0	1016.0	3104.0
Crystal size/mm^3	0.08 × 0.06 × 0.05	0.075× 0.04 × 0.03	0.25×0.02 × 0.02	0.06×0.04 × 0.02	0.25×0.025 × 0.025
Radiation	MoKα (λ=0.71073)	MoKα (λ=0.71073)	MoKα (λ=0.71073)	MoKα (λ=0.71073)	MoKα (λ=0.71073)
2Θ range for data collection/°	4.162 to 51.41	4.534 to 56.56	4.592 to 51.36	3.888 to 51.538	3.372 to 51.364
Index ranges	$-9 \le h \le 9$, $-12 \le k \le 12$, $-14 \le l \le 13$	$-8 \le h \le 8$, $-14 \le k \le 18$, $-16 \le l \le 15$	$-8 \le h \le 10$, $-10 \le k \le 10$, $-22 \le l \le 22$	$-13 \le h \le 14$, $-15 \le k \le 15$, $-17 \le l \le 17$	$-15 \le h \le 15$, $-36 \le k \le 36$, $-15 \le l \le 16$
Reflections collected	11656	8218	15289	34796	61895
Independent reflections	3491 [R_{int}= 0.0525, R_{sigma}= 0.0504]	2550 [R_{int}= 0.0194, R_{sigma}= 0.0208]	4464 [R_{int}= 0.0335, R_{sigma}= 0.0369]	7433 [R_{int}= 0.0666, R_{sigma}= 0.0508]	9572 [R_{int}= 0.1160, R_{sigma}= 0.0614]
Data/restraints/parameters	3491/0/147	2550/0/108	4464/0/221	7433/25/512	9572/264/477
Goodness-of-fit on F^2	1.060	1.005	1.049	1.031	1.077
Final R indexes [I>=2σ (I)]	R_1= 0.0262, wR_2= 0.0623	R_1= 0.0179, wR_2= 0.0424	R_1= 0.0396, wR_2= 0.1092	R_1= 0.0449, wR_2= 0.0827	R_1= 0.0999, wR_2= 0.2575
Final R indexes [all data]	R_1=0.0287, wR_2= 0.0638	R_1=0.0197, wR_2= 0.0432	R_1= 0.0434, wR_2= 0.1115	R_1= 0.0659, wR_2= 0.0895	R_1= 0.1152, wR_2= 0.2730
Largest diff. peak/hole / e Å^{-3}	1.34/-1.64	0.34/-0.65	0.84/-1.33	0.52/-0.38	2.85/-1.73

$R1 = \Sigma||F_o|-|F_c||/\Sigma|F_o|$, $wR2 = [\Sigma[w(F_o^2-F_c^2)^2]/\Sigma[w(F_o^2)^2]]^{1/2}$, $w = 1/[\sigma^2(F_o^2) + (aP)^2 + bP]$, where $P = [\max(F_o^2,0) + 2F_c^2]/3$

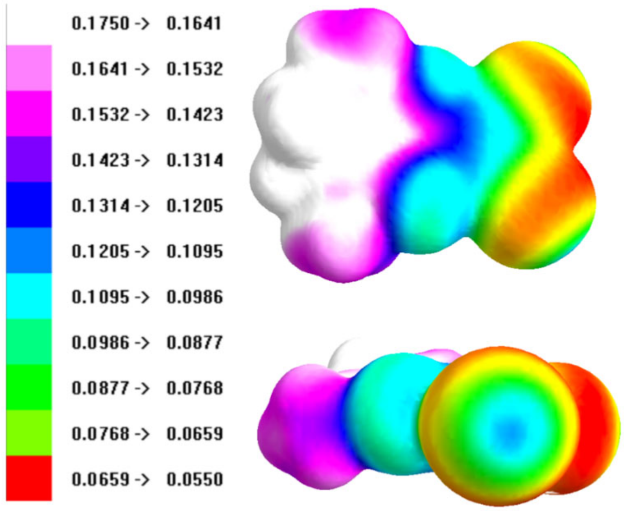

Figure S1. Electrostatic potential mapped onto the 0.0025 isovalue of the electronic density for complex **1'**.

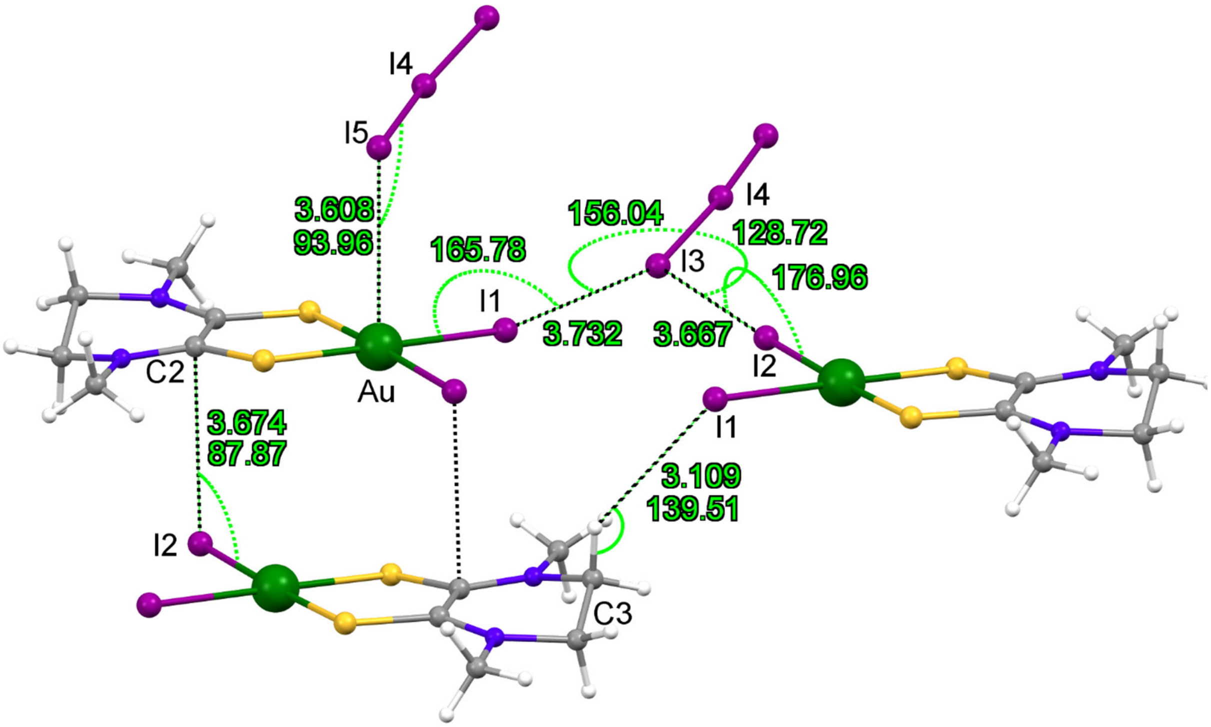

Figure S2. Portion of the crystal packing of **1** with intermolecular interactions depicted ad dashed bonds. Angles (°) and distances (Å) are indicated.

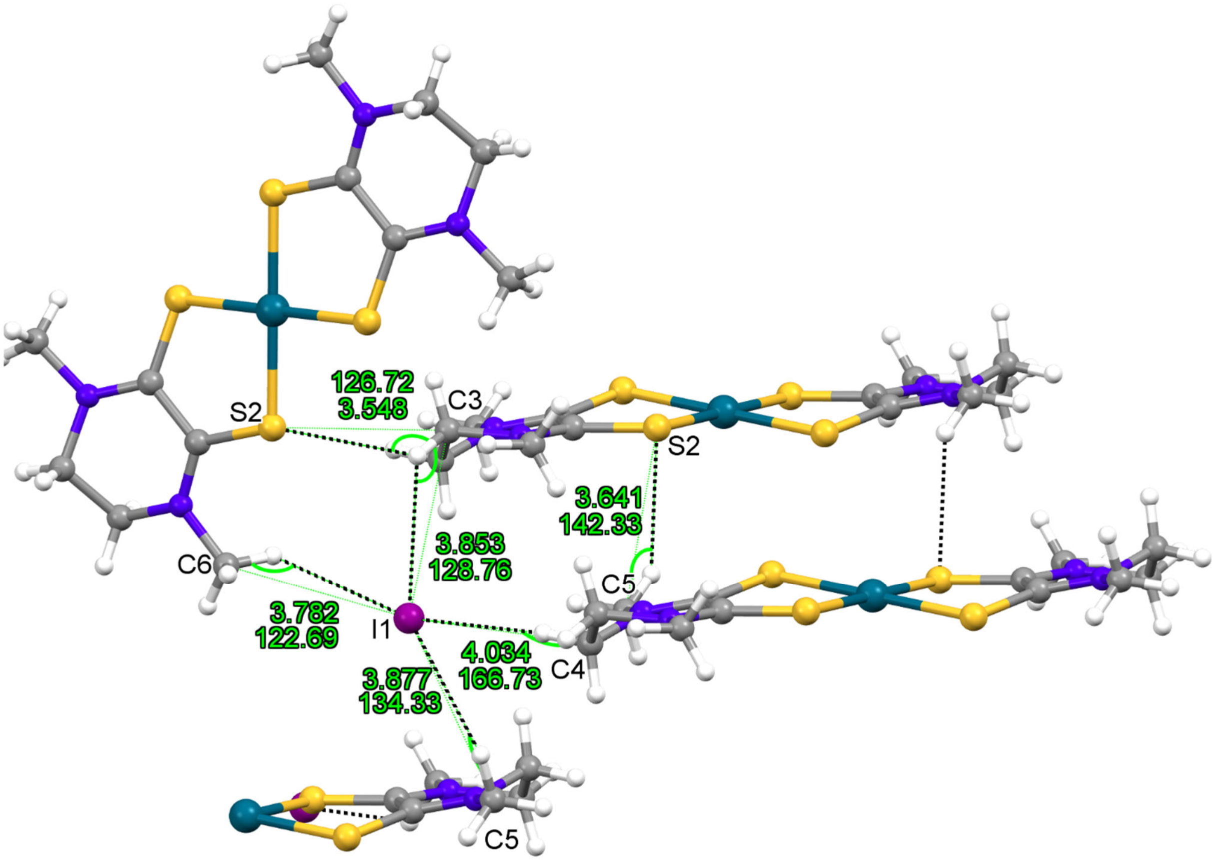

Figure S3. Portion of the crystal packing of **2a** with intermolecular interactions depicted ad dashed bonds. Angles (°) and distances (Å) are indicated.

Table S2. Dihedral angles between the mean planes (**A** and **B**) of the thioamido groups within each ligand.

Complex	Angle (°)
$[Au(Me_2pipdt)I_2]I_3$	8.7
$[Pd(Me_2pipdt)_2](I)_2$	11.3
$[Cu(Me_2pipdt)_2]I_3$	20.1; 17.1
$[Cu(Me_2pipdt)_2]BF_4$*	15.9; 26.8; 9.1; 20.4
$[Cu(Me_2dazdt)_2]I_3$*	53.7; 55.4; 54.6;58.1

* two complex molecules are present in the asymmetric unit.

Table S3. Dihedral angles between the mean planes (**I** and **II**) of the coordination environment.

Complex	Plane I	Plane II	Angle (°)
[Au(Me$_2$pipdt)I$_2$]I$_3$	S2C2C1S1Au	I2AuI1	3.69
[Pd(Me$_2$pipdt)$_2$](I)$_2$	S2C2C1S1Pd	S2C2C1S1Pd[a]	0
[Cu(Me$_2$pipdt)$_2$]I$_3$	S11C11C21S21Cu	S12C12C22S22Cu	64.23
[Cu(Me$_2$pipdt)$_2$]BF$_4$*	S11C11C21S21Cu1	S12C12C22S22Cu1	84.69
	S13C13C23S23Cu2	S14C14C24S24Cu2	74.24
[Cu(Me$_2$dazdt)$_2$]I$_3$*	S11C11C21S21Cu1	S12C12C22S22Cu1	79.99
	S13C13C23S23Cu2	S14C14C24S24Cu2	79.97

* two complex molecules are present in the asymmetric unit. [a] Symmetry generated atoms

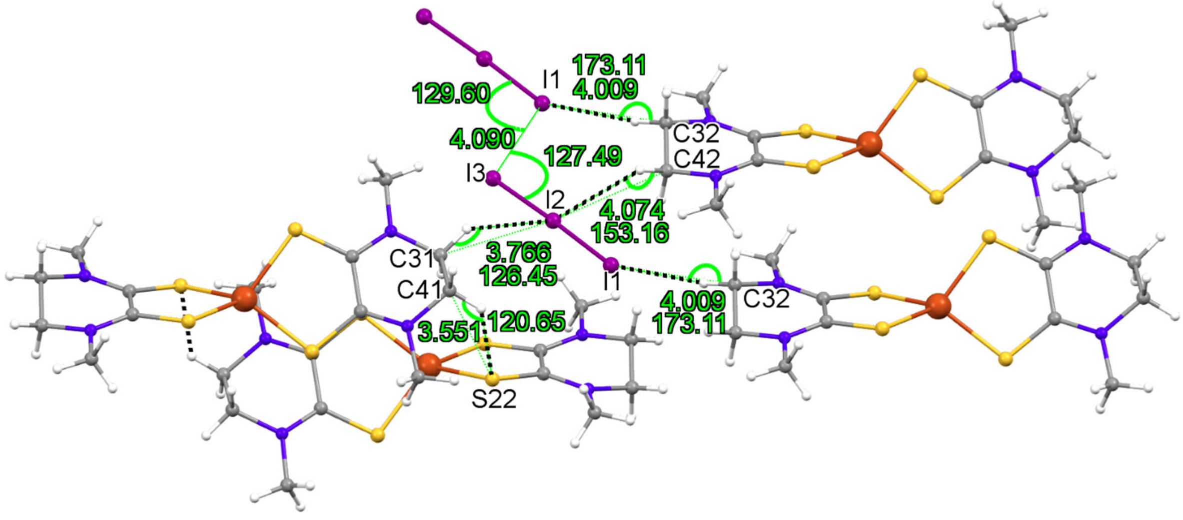

Figure S4. Portion of the crystal packing of **3** with intermolecular interactions depicted ad dashed bonds. Angles (°) and distances (Å) are indicated.

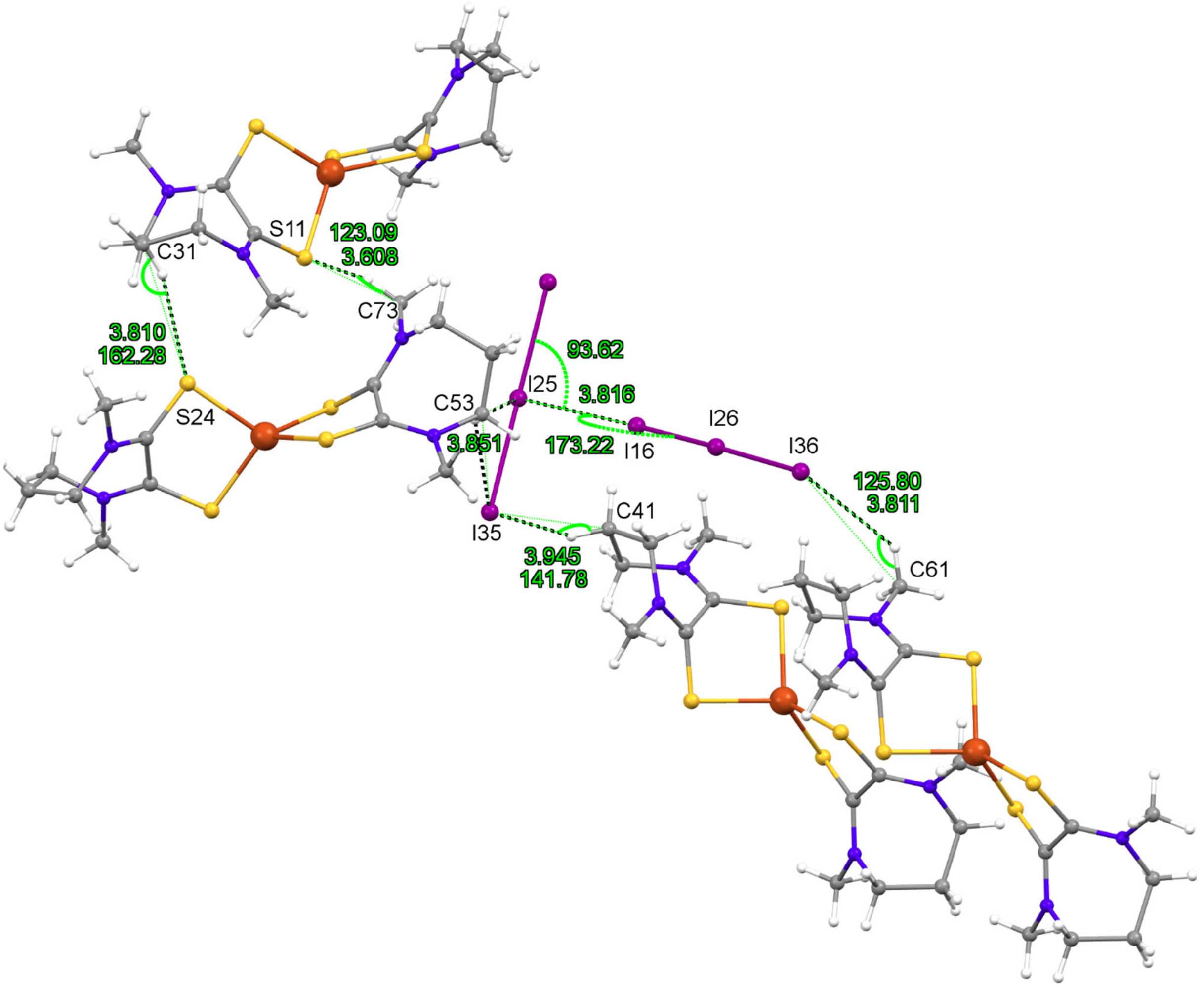

Figure S5. Portion of the crystal packing of **5** with intermolecular interactions depicted ad dashed bonds. Angles (°) and distances (Å) are indicated.

S2. Vibrational spectroscopy

Table S4. Most significant vibrational peaks for **1**, **2**, **3** and **5**.

	v(CN)	v_{sym}(I–I–I)	v_{asym}(I–I–I)<br>*or v*(I–I)
Me_2pipdt	1503vs		
[AuI_2(Me_2pipdt)]I_3	1552vs	101ms	139-sh<br>162s, 150vs
[Pd(Me_2pipdt)$_2$]I_6	1538vs	109vs	133s, 147s
[Cu(Me_2pipdt)$_2$]I_3	1512vs	108vs	140s
Me_2dazdt	1493vs		
[Cu(Me_2dazdt)$_2$]I_3	1513vs	111vs	135m

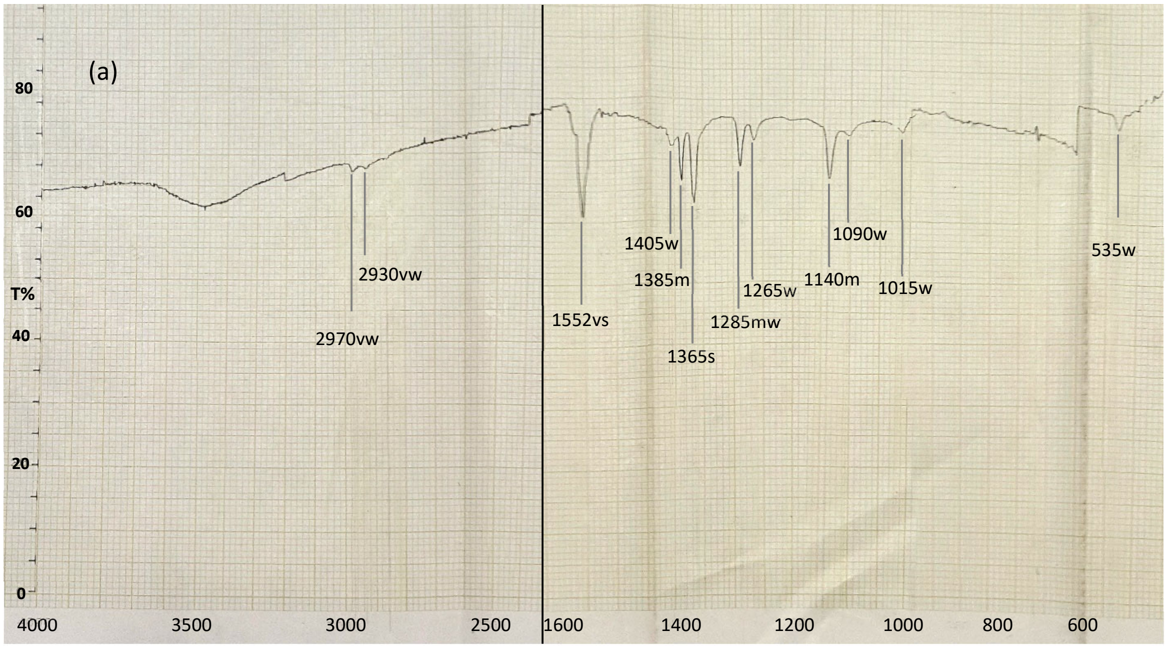

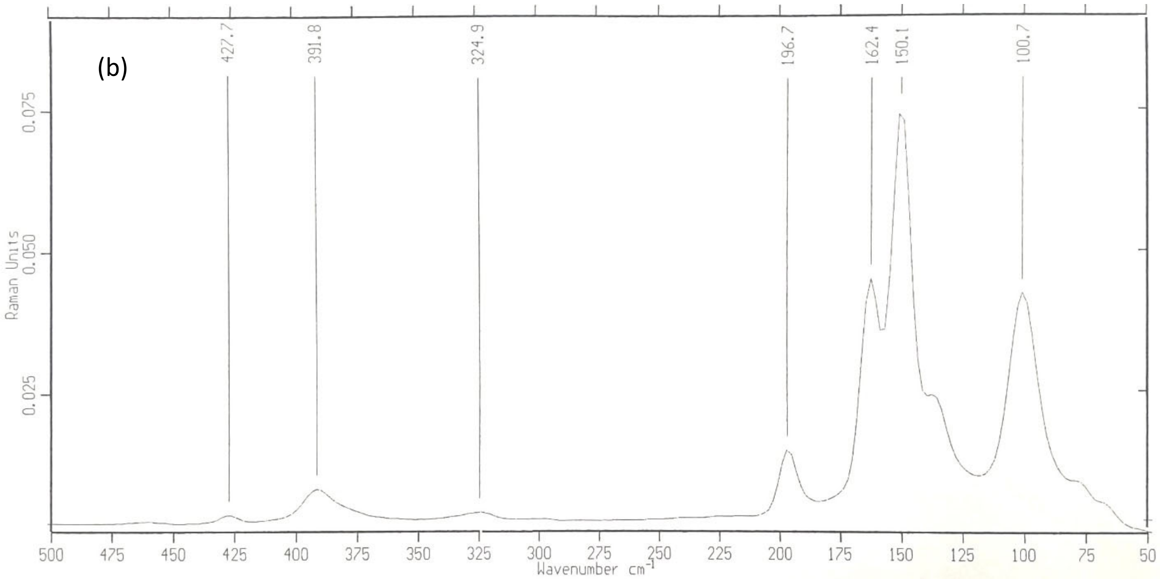

Figure S6. Vibrational spectroscopy characterization of **1**: IR (**a**, 4000-400cm^{-1}) and FT-Raman (**b**, 500-50cm^{-1}).

Zoom A

Zoom B

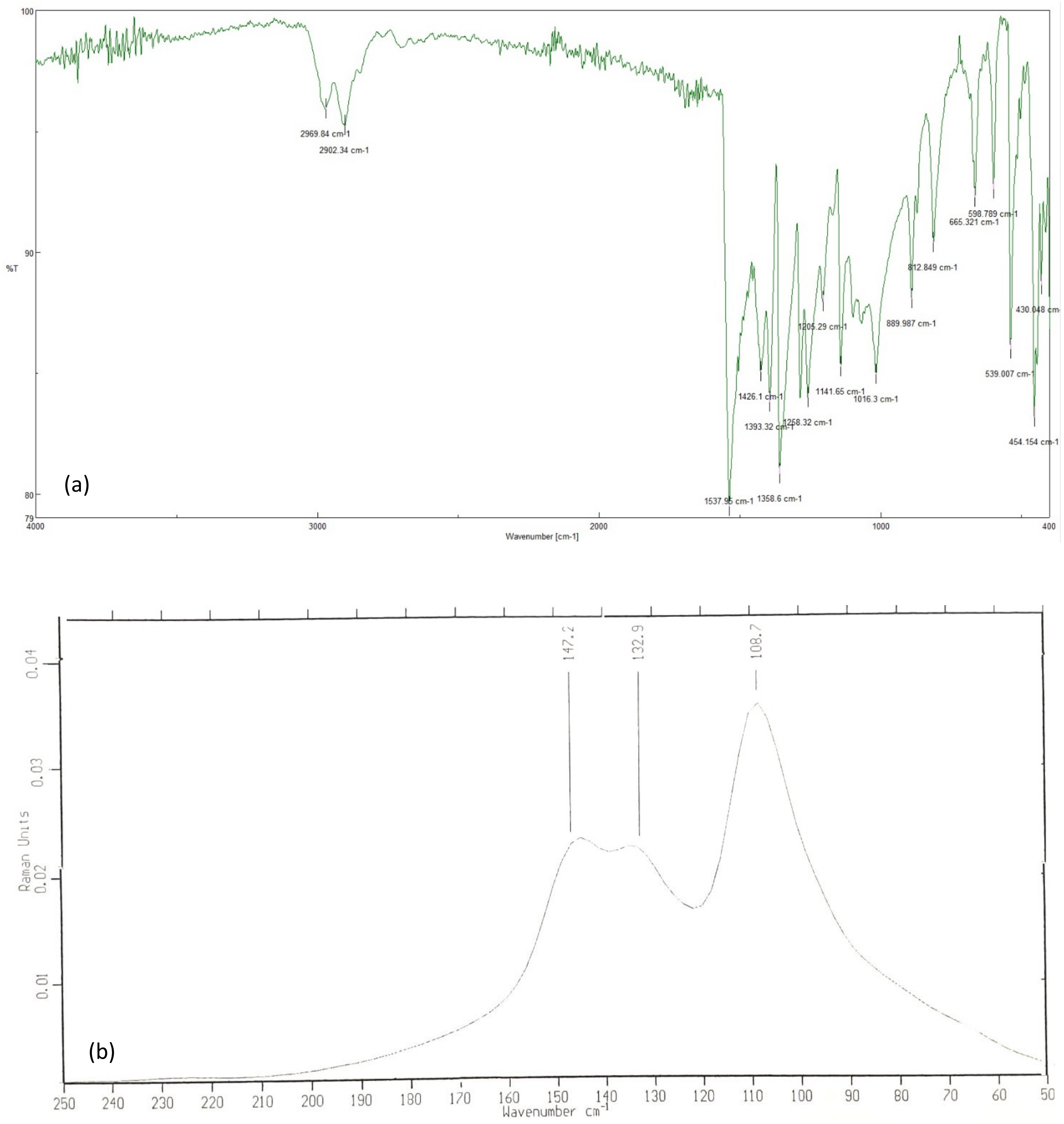

Figure S7. Vibrational spectroscopy characterization of **2**: FT-IR **(a**, 4000-400cm^{-1}**)** and FT-Raman **(b**, 250-50cm^{-1}**)**.

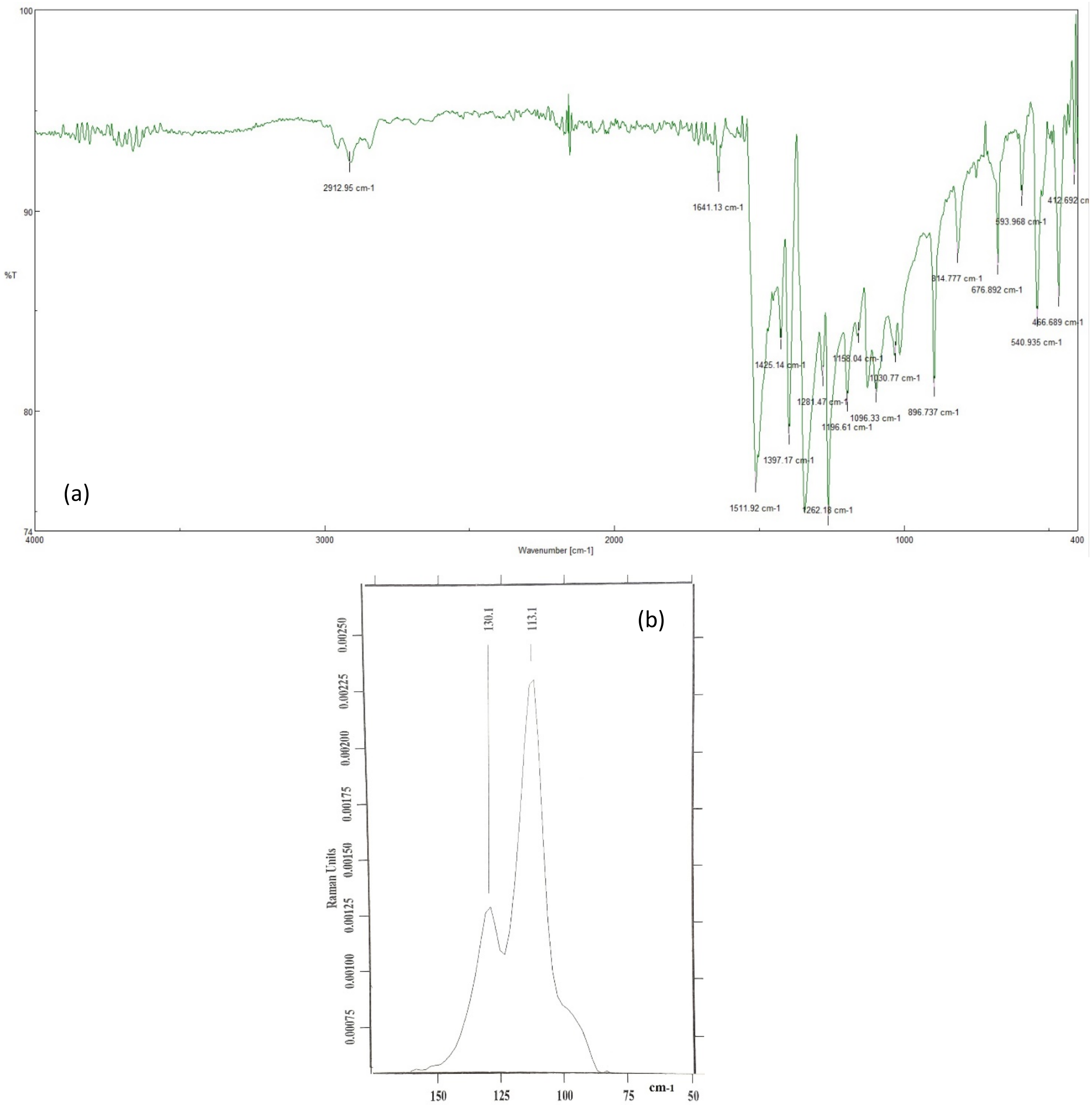

Figure S8. Vibrational spectroscopy characterization of **3**: FT-IR **(a**, 4000-400cm^{-1}**)** and FT-Raman **(b**, 200-50cm^{-1}**)**.

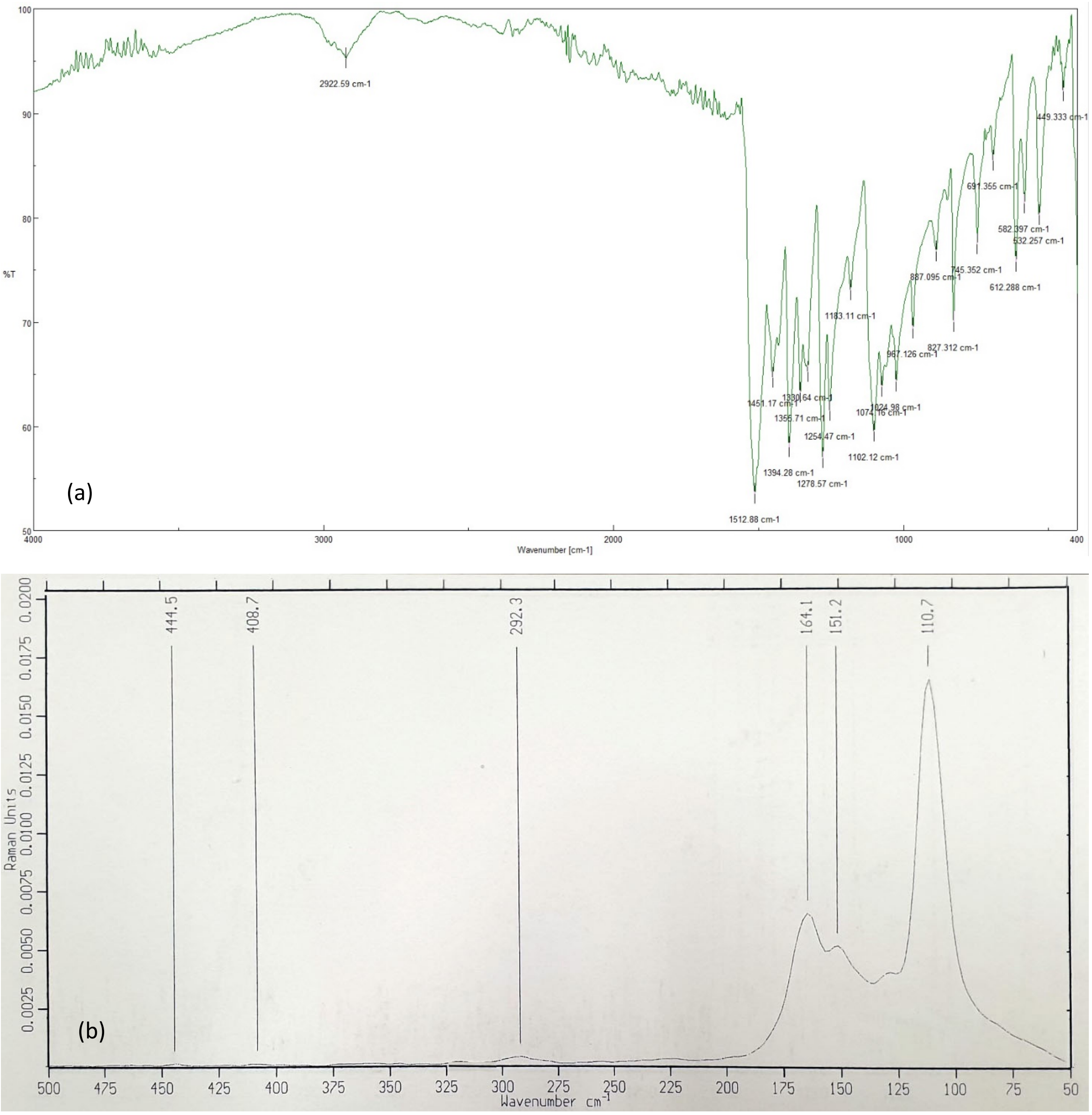

Figure S9. Vibrational spectroscopy characterization of **5**: FT-IR **(a**, 4000-400cm^{-1}**)** and FT-Raman **(b**, 500-50cm^{-1}**)**.

S3. DFT calcultions

Table S5. Comparison of crystallographic and *calculated* bond distances (Å) and angles (°) for compounds $[Au(Me_2pipdt)I_2]^+$ and $[Pd(Me_2pipdt)_2]^{2+}$.

$[Au(Me_2pipdt)I_2]^+$		$[Pd(Me_2pipdt)_2]^{2+}$	
Au-S(1)	2.316(2) (*2.420*)	Pd-S(1)	2.2952(5) (*2.343*)
Au-S(2)	2.319(2) (*2.420*)	Pd-S(2)	2.2826(5) (*2.343*)
Au-I(1)	2.6113(4) (*2.617*)	C(1)-S(1)	1.700(2) (*1.707*)
Au-I(2)	2.6139(5) (*2.617*)	S(2)-C(2)	1.701(2) (*1.707*)
C(1)-S(1)	1.694(5) (*1.706*)	C(1)-N(1)	1.313(2) (*1.328*)
C(2)-S(2)	1.699(6) (*1.706*)	C(2)-N(2)	1.307(2) (*1.328*)
C(1)-N(1)	1.311(7) (*1.332*)	C(1)-C(2)	1.500(3) (*1.500*)
C(2)-N(2)	1.319(7) (*1.332*)		
C(1)-C(2)	1.493(7) (*1.506*)		
S(1)-Au-S(2)	89.97(5) (*86.98*)	S(1)-Pd-S(2)	88.58(2) (*87.62*)

Table S6. Comparison of crystallographic and *calculated* bond distances (Å) and angles (°) for compounds $[Cu(Me_2pipdt)_2]^+$ and $[Cu(Me_2dazdt)_2]^+$.

$[Cu(Me_2pipdt)_2]^+$		$[Cu(Me_2dazdt)_2]^+$	
Cu-S(11)	2.272(2) (*2.391*)	Cu(1)-S(11)	2.342(4) (*2.440*)
Cu-S(21)	2.295(2) (*2.392*)	Cu(1)-S(21)	2.344(4) (*2.443*)
C(11)-S(11)	1.689(5) (*1.685*)	C(11)-S(11)	1.69(1) (*1.69*)
C(21)-S(21)	1.679(6) (*1.685*)	C(21)-S(21)	1.69(2) (*1.69*)
C(11)-N(11)	1.323(7) (*1.346*)	C(11)-N(11)	1.29(2) (*1.34*)
C(21)-N(21)	1.322(7) (*1.346*)	C(21)-N(21)	1.34(2) (*1.34*)
C(11)-C(21)	1.515(8) (*1.521*)	C(11)-C(21)	1.49(2) (*1.52*)
Cu-S(12)	2.276(2) (*2.392*)	Cu(1)-S(12)	2.309(4) (*2.441*)
Cu-S(22)	2.283(2) (*2.391*)	Cu(1)-S(22)	2.338(4) (*2.441*)
C(12)-S(12)	1.679(5) (*1.685*)	C(12)-S(12)	1.69(1) (*1.69*)
C(22)-S(22)	1.670(5) (*1.685*)	C(22)-S(22)	1.69(1) (*1.69*)
C(12)-N(12)	1.313(7) (*1.346*)	C(12)-N(12)	1.29(2) (*1.34*)
C(22)-N(22)	1.318(7) (*1.346*)	C(22)-N(22)	1.31(2) (*1.34*)
C(12)-C(22)	1.523(8) (*1.521*)	C(12)-C(22)	1.52(2) (*1.52*)
S(11)-Cu-S(21)	91.71(6) (*88.65*)	S(11)-Cu(1)-S(21)	93.1(1) (*90.55*)
S(12)-Cu-S(22)	91.24(5) (*88.65*)	S(12)-Cu(1)-S(22)	93.6(1) (*90.53*)

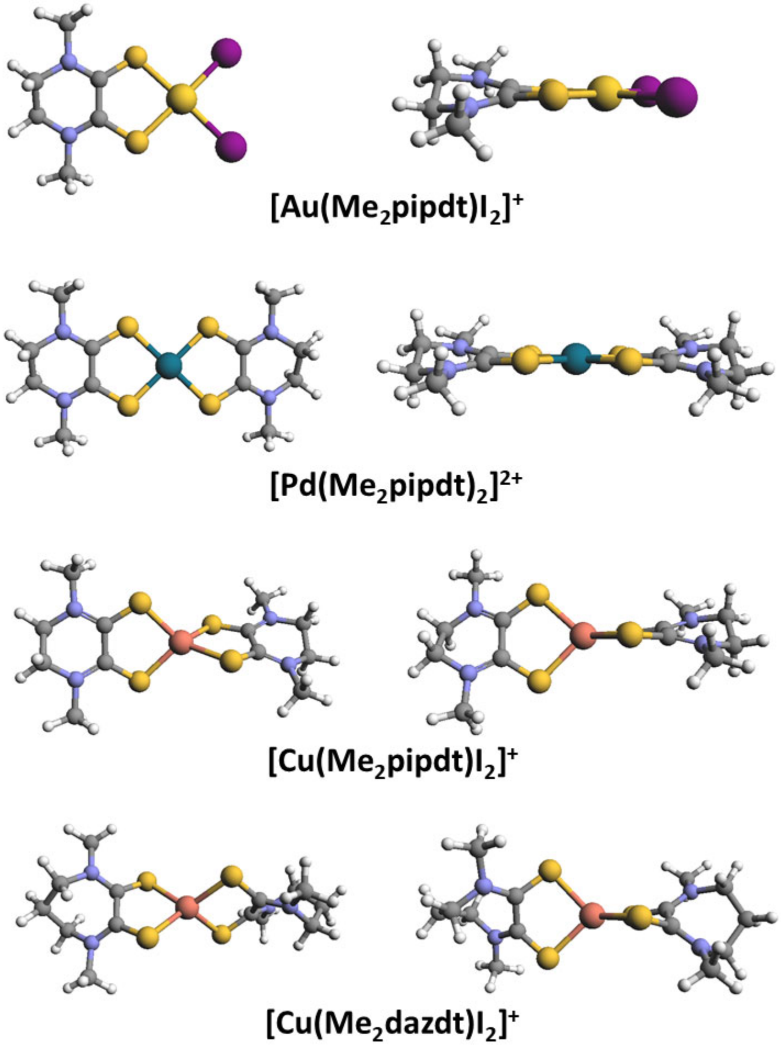

Figure S10. Gas-phase DFT optimized geometries for **1’**, **2’**, **3’** and **5’**.

LUMO+2	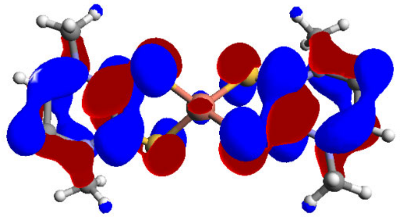
LUMO+1	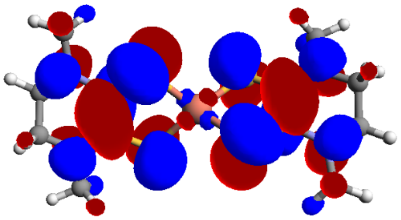
**LUMO**	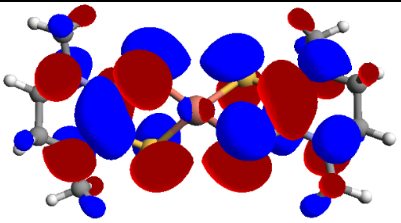
**HOMO**	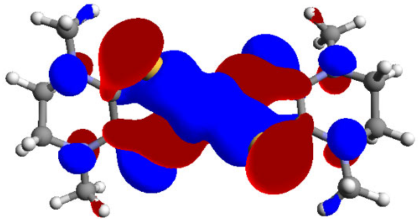
HOMO–1	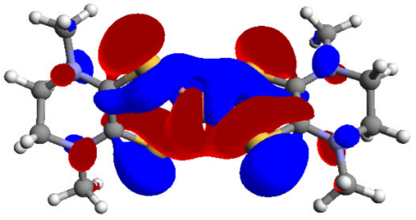
HOMO–2	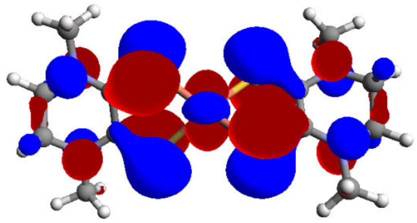

Figure S11. DFT calculated molecular orbitals of complex **3’** (isovalue plot 0.02).

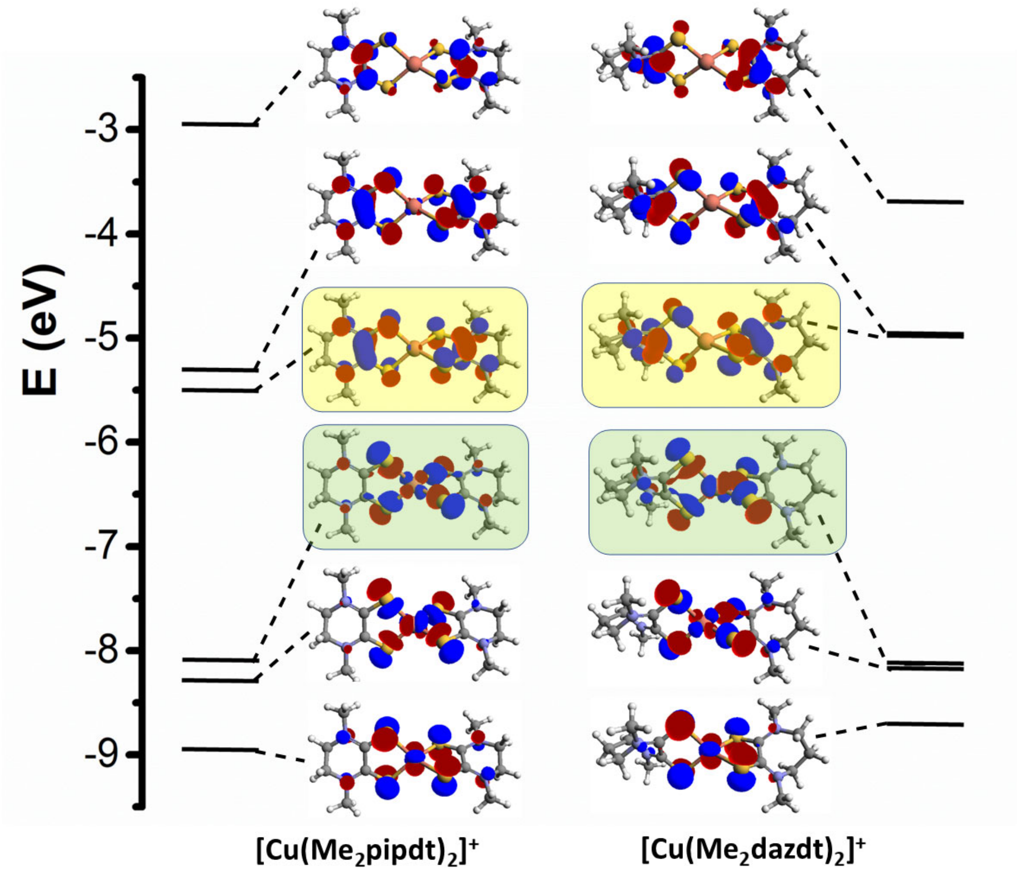

Figure S12. DFT calculated molecular orbitals for complexes **3’** and **5’**. Starting from the upper part, the figure depicts all the MO ranging from the LUMO+2 to the HOMO–2; for each complex, HOMO and LUMO are highlighted in pale green and yellow colour, respectively (isovalue = 0.04).

S4. ^{1}H and ^{13}C NMR spectra

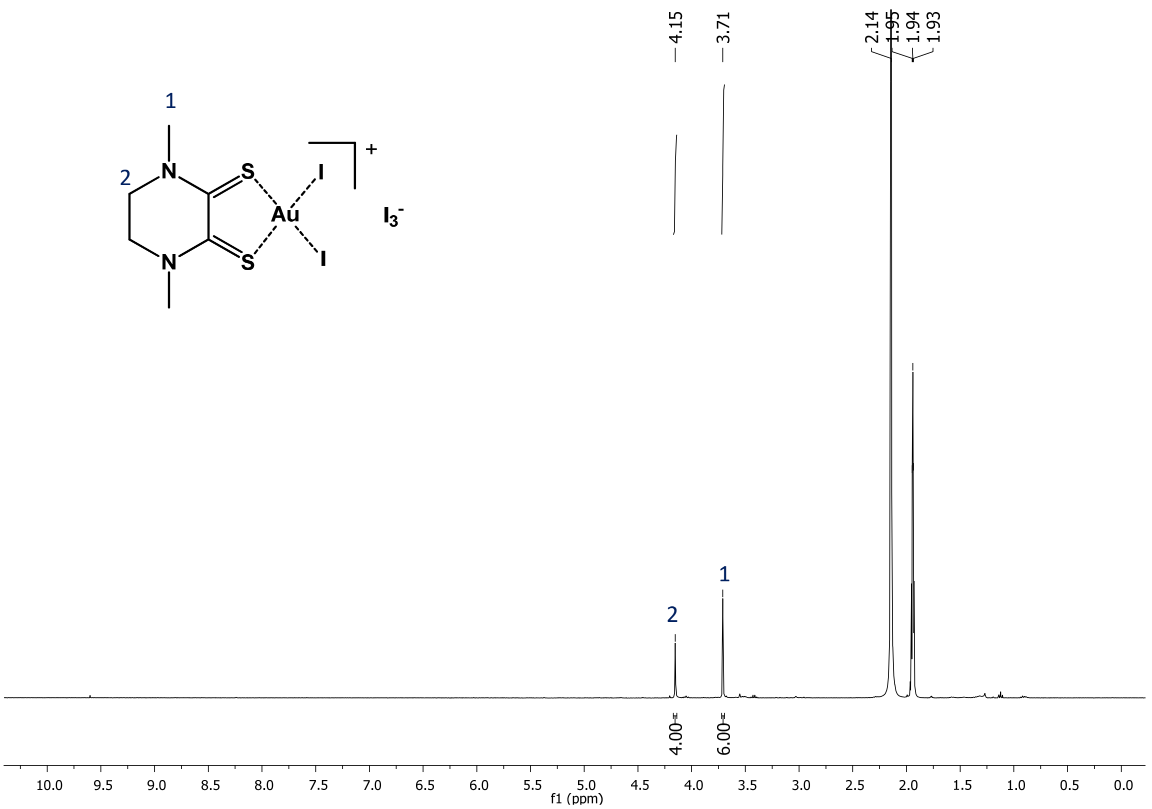

Figure S13. ^{1}H-NMR (400 MHz, CD_3CN) spectrum of $[Au(Me_2pipdt)I_2]I_3$ (**1**). Compound **1** was sparingly soluble in CD_3CN or DMSO-d^6.

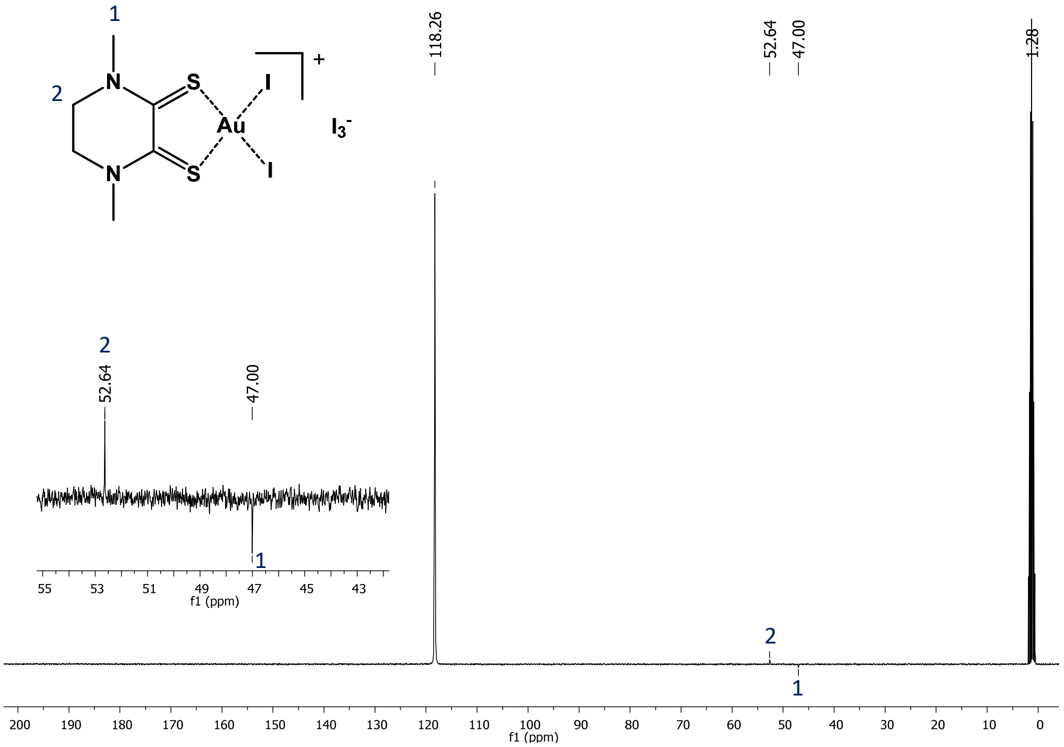

Figure S14. ^{13}C- deptq135 NMR (100 MHz, CD_3CN) spectrum of $[Au(Me_2pipdt)I_2]I_3$ (**1**). Compound **1** was sparingly soluble in CD_3CN or DMSO-d^6.

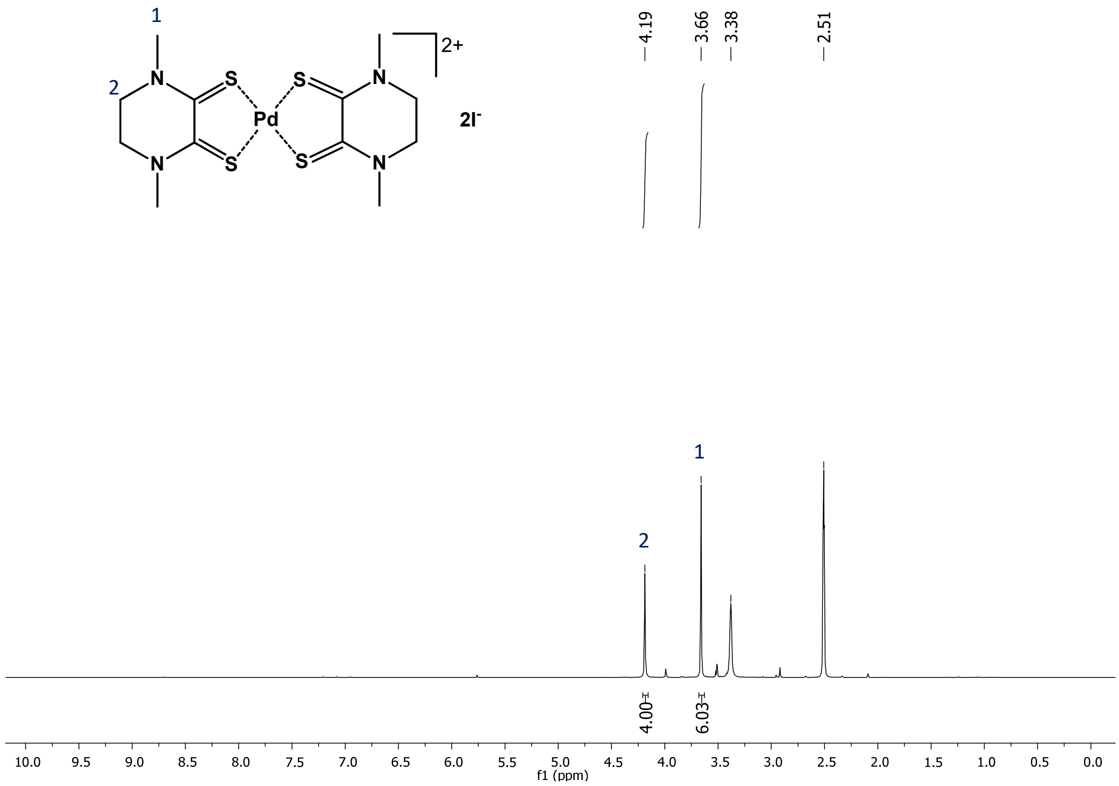

Figure S15. ^{1}H-NMR (400 MHz, DMSO-d^{6}) spectrum of [Pd(Me_2pipdt$_2$](I)$_2$ (**2a**).

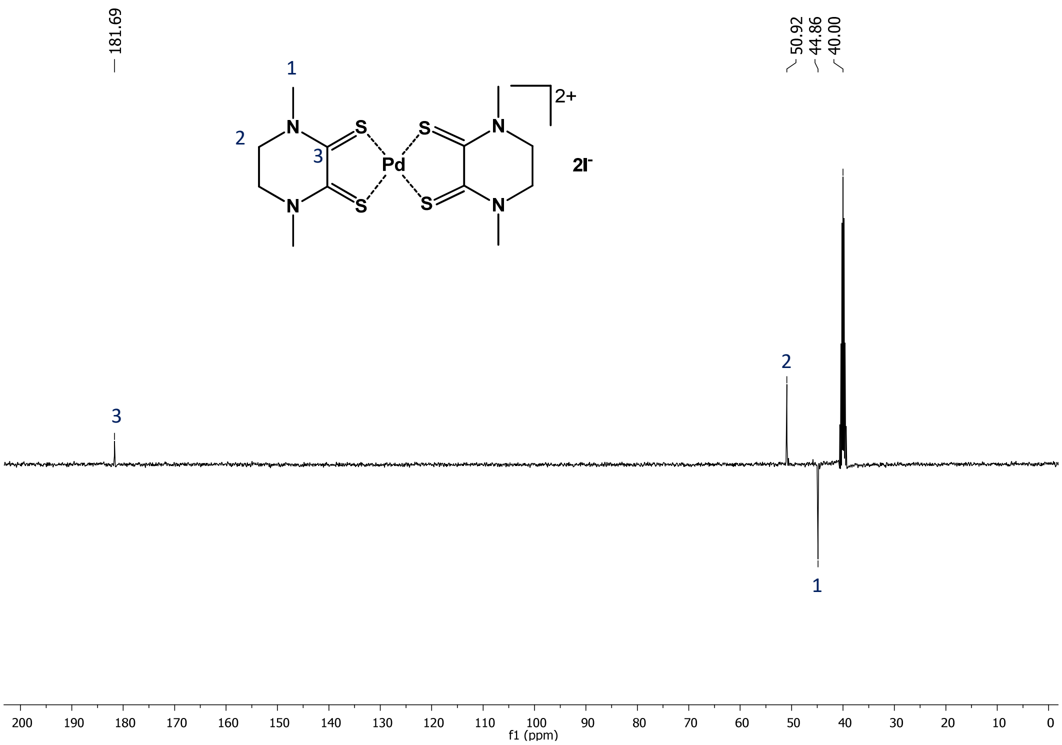

Figure S16. ^{13}C-deptq135 NMR (100 MHz, DMSO-d^{6}) spectrum of [Pd(Me_2pipdt$_2$](I)$_2$ (**2a**).

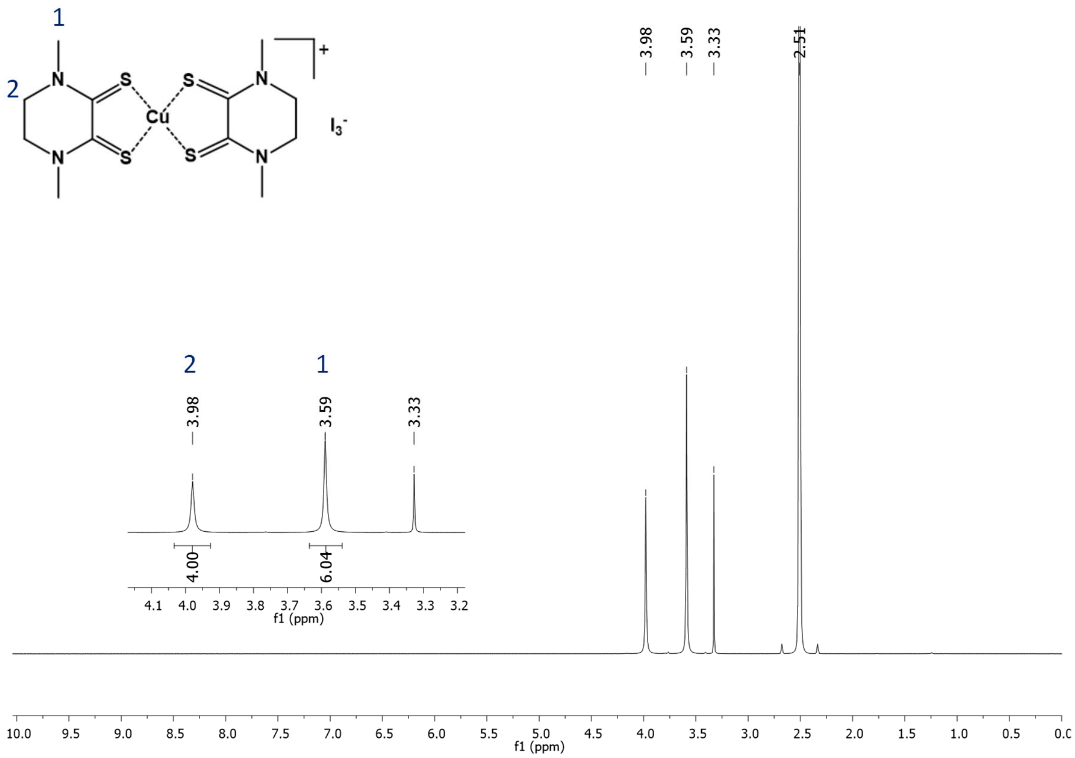

Figure S17. ^{1}H-NMR (400 MHz, DMSO-d[6]) spectrum of [Cu(Me_2pipdt)$_2$]I_3 (**3**).

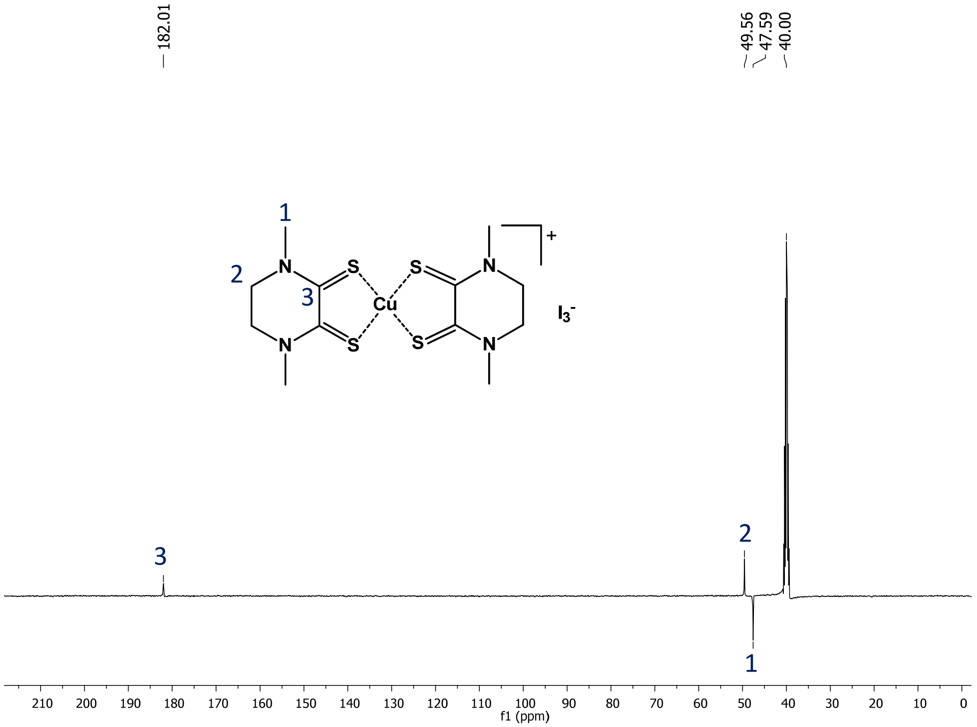

Figure S18. ^{13}C-deptq135 NMR (100 MHz, DMSO-d[6]) spectrum of [Cu(Me_2pipdt)$_2$]I_3 (**3**).

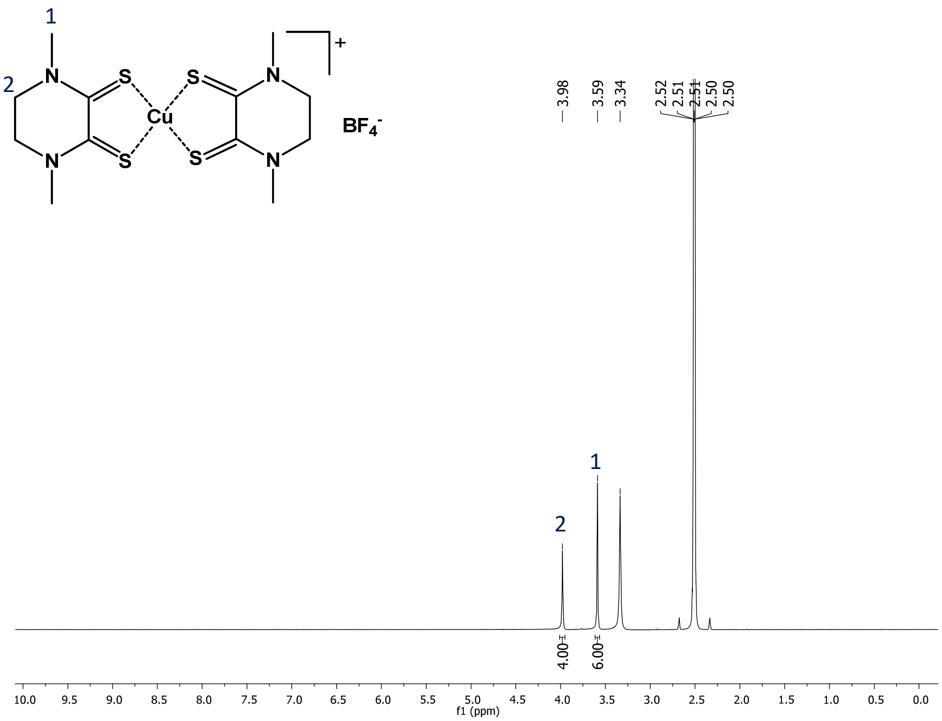

Figure S19. ^{1}H-NMR (400 MHz, DMSO-d^{6}) spectrum of [Cu(Me$_2$pipdt)$_2$]BF$_4$ (**4**).

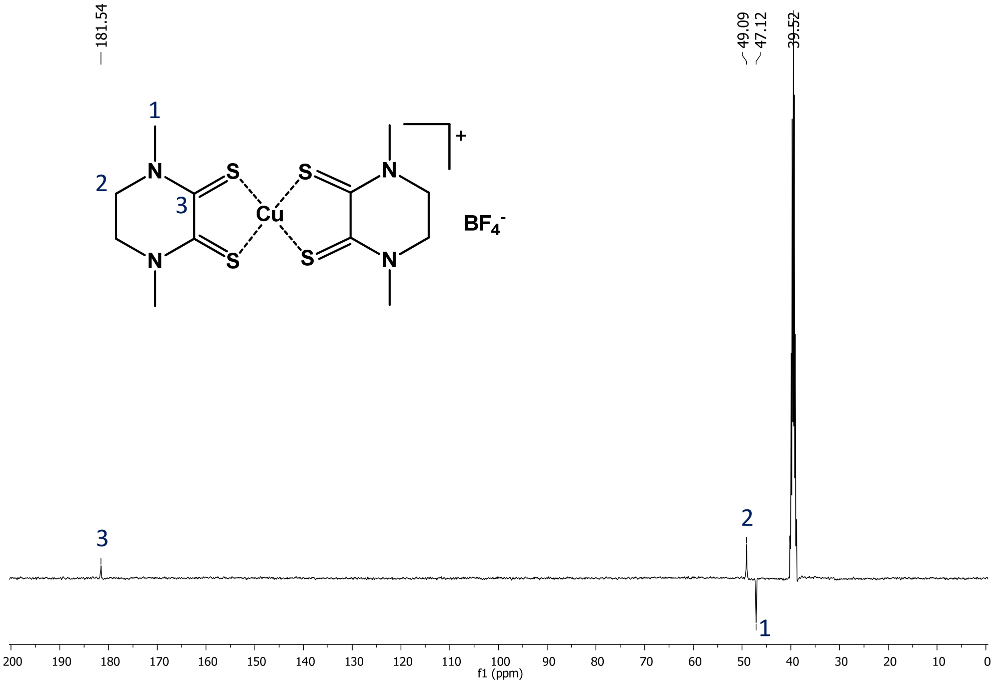

Figure S20. ^{13}C-deptq NMR (100 MHz, DMSO-d^{6}) spectrum of [Cu(Me$_2$pipdt)$_2$]BF$_4$ (**4**).

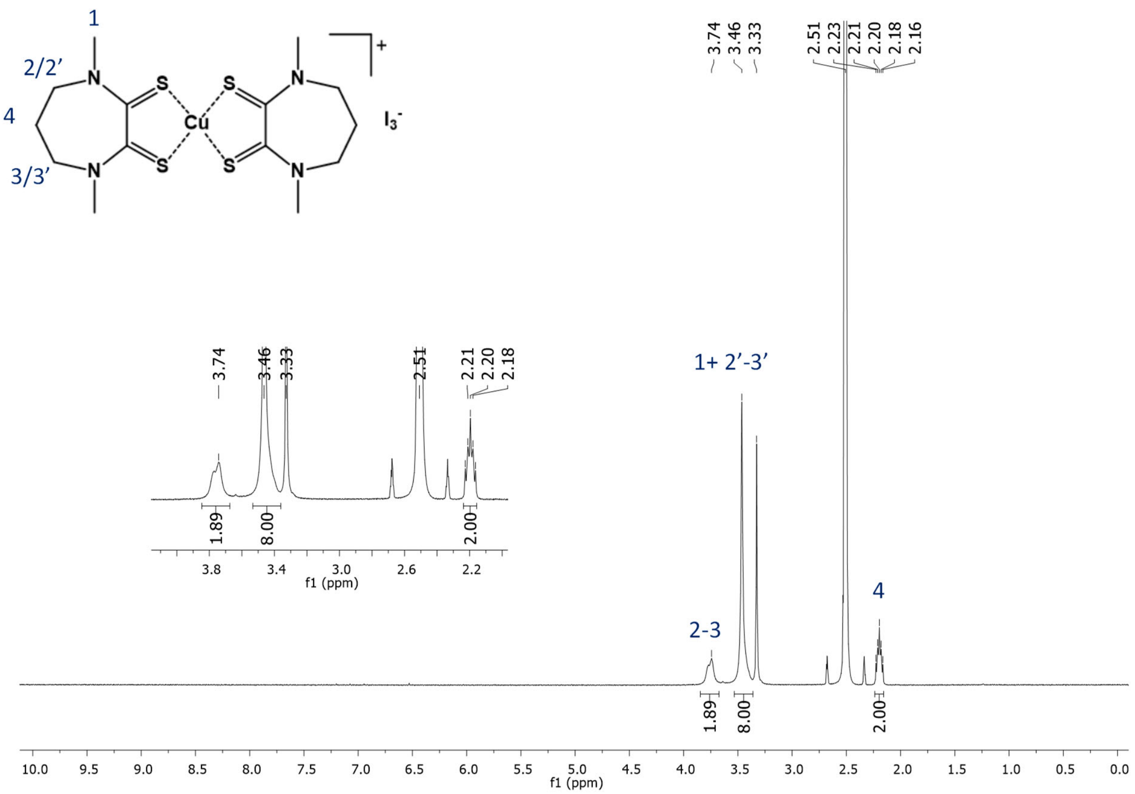

Figure S21. ^{1}H-NMR (400 MHz, DMSO-d[6]) spectrum of [Cu(Me$_2$dazdt)$_2$]I$_3$ (**5**).

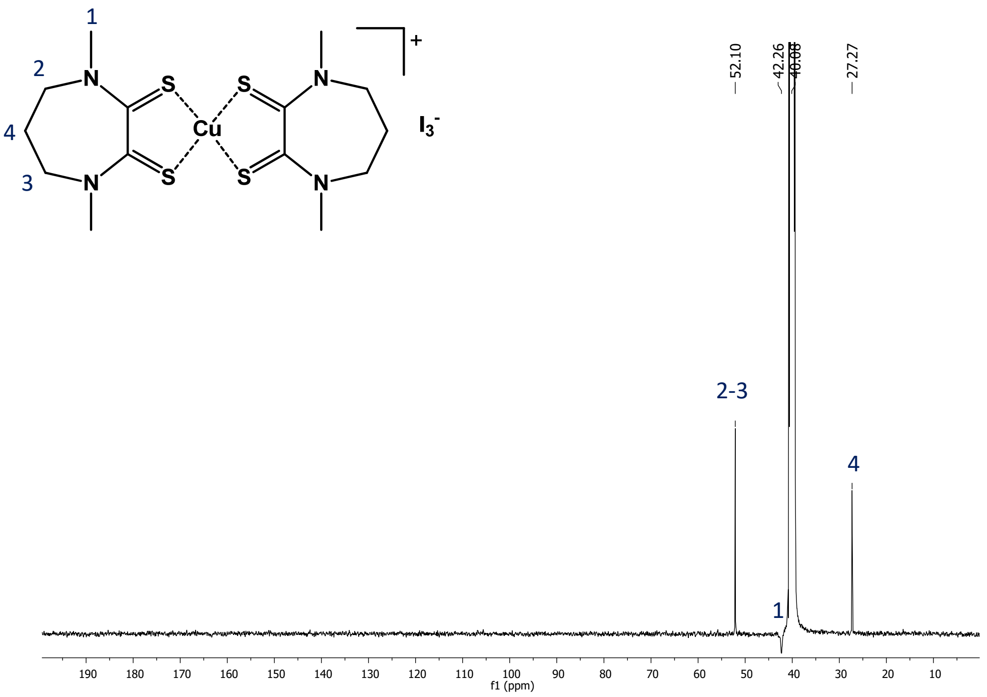

Figure S22. ^{13}C-deptq135 NMR (100 MHz, DMSO-d[6]) spectrum of [Cu(Me$_2$dazdt)$_2$]I$_3$ (**5**).

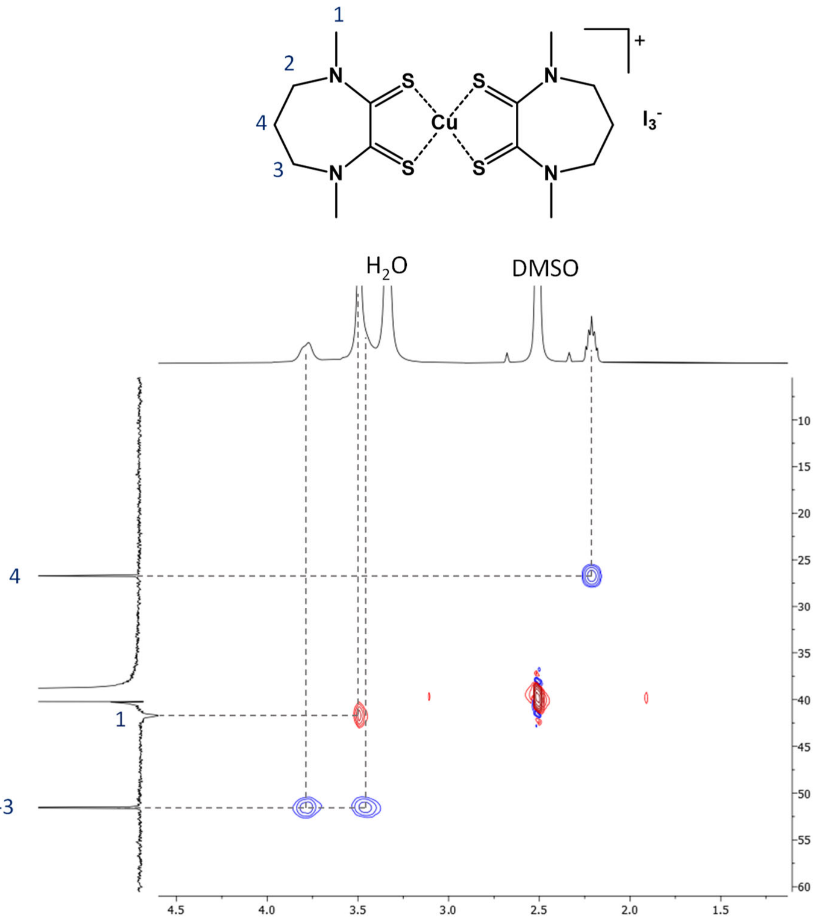

Figure S23. ^{1}H-^{13}C HSQC (DMSO-d^6) spectrum of [Cu(Me$_2$dazdt)$_2$]I$_3$ (**5**). The ^{13}C-deptq135 1D trace was used for the ^{13}C dimension.
